# Substrate-Dependent Fermentation of Bamboo in Giant Panda Gut Microbiomes: Leaf Primarily to Ethanol and Pith to Lactate

**DOI:** 10.3389/fmicb.2020.00530

**Published:** 2020-03-31

**Authors:** Alberto Scoma, Way Cern Khor, Marta Coma, Robert Heyer, Ruben Props, Jonas Schoelynck, Tim Bouts, Dirk Benndorf, Desheng Li, Hemin Zhang, Korneel Rabaey

**Affiliations:** ^1^Center for Microbial Ecology and Technology, University of Ghent, Ghent, Belgium; ^2^Department of Bioscience, Microbiology Section, Aarhus University, Aarhus C, Denmark; ^3^Department of Engineering, Biological and Chemical Engineering, Aarhus University, Aarhus N, Denmark; ^4^Bioprocess Engineering, Otto von Guericke University of Magdeburg, Magdeburg, Germany; ^5^Department of Biology, University of Antwerp, Antwerp, Belgium; ^6^Pairi Daiza Foundation, Brugelette, Belgium; ^7^Bioprocess Engineering, Max Planck Institute for Dynamics of Complex Technical Systems, Magdeburg, Germany; ^8^China Conservation and Research Centre for Giant Panda, Dujiangyan City, China

**Keywords:** giant panda, cellulose, hemicellulose, alpha amylase, lignocellulose, fermentation, gut microbiome, ethanol

## Abstract

The giant panda is known worldwide for having successfully moved to a diet almost exclusively based on bamboo. Provided that no lignocellulose-degrading enzyme was detected in panda’s genome, bamboo digestion is believed to depend on its gut microbiome. However, pandas retain the digestive system of a carnivore, with retention times of maximum 12 h. Cultivation of their unique gut microbiome under controlled laboratory conditions may be a valid tool to understand giant pandas’ dietary habits, and provide valuable insights about what component of lignocellulose may be metabolized. Here, we collected gut microbiomes from fresh fecal samples of a giant panda (either entirely green or yellow stools) and supplied them with green leaves or yellow pith (i.e., the peeled stem). Microbial community composition was substrate dependent, and resulted in markedly different fermentation profiles, with yellow pith fermented to lactate and green leaves to lactate, acetate and ethanol, the latter to strikingly high concentrations (∼3%, v:v, within 3.5 h). Microbial metaproteins pointed to hemicellulose rather than cellulose degradation. The alpha-amylase from the giant panda (E.C. 3.2.1.1) was the predominant identified metaprotein, particularly in reactors inoculated with pellets derived from fecal samples (up to 60%). Gut microbiomes assemblage was most prominently impacted by the change in substrate (either leaf or pith). Removal of soluble organics from inocula to force lignocellulose degradation significantly enriched *Bacteroides* (in green leaf) and *Escherichia*/*Shigella* (in yellow pith). Overall, different substrates (either leaf or pith) markedly shaped gut microbiome assemblies and fermentation profiles. The biochemical profile of fermentation products may be an underestimated factor contributing to explain the peculiar dietary behavior of giant pandas, and should be implemented in large scale studies together with short-term lab-scale cultivation of gut microbiomes.

## Introduction

The iconic giant panda (*Ailuropoda melanoleuca*) is known worldwide for being a carnivore (belonging to the family of the *Ursidae*, thus a bear) that moved to a vegetarian diet based almost exclusively on bamboo, a member of the grass family *Poaceae* [99% in the wild (Schaller et al., 1985)]. Paleontological and molecular evidence suggest this dietary shift initiated about 2 million years ago (Jin et al., 2007; Zhao et al., 2010). While dentition, skull and jaw musculature adapted to crush and grind the hard bamboo culms, surprisingly the soft tissues of the gut remained those typical of its carnivorous ancestors (Wildt et al., 2006). The latter is considered the main reason for the short retention time of ingested bamboo in the gastrointestinal tract (Dierenfeld et al., 1982), which has no apparent special compartment to retain food contrary to monogastric herbivores and ruminants. Clearance of the digesta is generally observed between 5 and 12 h (Dierenfeld et al., 1982; Mainka et al., 1989; Edwards et al., 2006; Senshu et al., 2007), although longer times have been reported in pandas with intestinal disorders (Mainka et al., 1989; Senshu et al., 2007) or depending on the portion of bamboo ingested [about 8 h for shoots, 10 h for stems and 14 h for leaves (Schaller et al., 1985)]. The low digestibility of bamboo by pandas [<40% on either a dry matter or apparent energy conversion basis (Dierenfeld et al., 1982; Senshu et al., 2007; Sims et al., 2007; Finley et al., 2011)], raised some questions over the evolutionary advantage of moving to such a diet (Gittleman, 1994). How giant pandas meet their nutritional and energetic requirements has not been fully resolved, particularly when compared to other large herbivores. Recent reports suggest that giant pandas are macronutritional carnivores (Nie et al., 2019), with the low efficiency of bamboo degradation compensated by maintaining a high daily feed intake [6 to 15% of body weight dry matter (Mainka et al., 1989; Dierenfeld, 1997)], constraining pandas to spend up to one third of their day in feeding (Edwards et al., 2006). In this context, short gut retention times and low energy expenditure of daily giant panda’s life are considered an advantage (Nie et al., 2015). The possibility that giant pandas feed on the more readily available bamboo cellular content, rather than on fermentation of its cell wall, was proposed (Dierenfeld et al., 1982; Senshu et al., 2014).

Pandas are extremely selective upon choosing which bamboo component to eat (i.e., shoots, leaves, branches, or stem) (Dierenfeld et al., 1982; Mainka et al., 1989; Edwards et al., 2006). Leaves are generally consumed all year long, however, a typical peak in stems consumption between March and May has been observed (Hansen et al., 2010; Williams et al., 2013; Knott et al., 2017) in both captive and wild pandas (Schaller et al., 1985; Taylor and Zisheng, 1987; Rybiski Tarou et al., 2005). As bamboo is an evergreen plant with leaves available throughout the year, the exact biological mechanisms motivating a dietary shift to stems remains unexplained. Stools color is testament of pandas’ selectiveness for different portions of bamboo, as even within the same day pandas may produce stools uniquely constituted of green shoots and leaves, along with others uniquely made of yellow pith (i.e., the peeled culm). Removal of the waxy outer cover from culms to get access to the softer, yellow pith before ingestion is a common behavior also in the wild (Schaller et al., 1985), and possibly linked to bamboo composition. A present assumption considers seasonal variation in bamboo nutrient content a reason for pandas’ dietary behavior (Knott et al., 2017). However, it appears counterproductive to forage on the less digestible, woody pith during the panda’s breeding season (March to April), when extra energy is spent to seek mates (Wildt et al., 2006).

Provided that no enzyme for the degradation of lignocellulose was found in giant panda’s genome (Li et al., 2010), it is assumed that the panda’s gut microbiome may fulfill this function. The essential contribution of gut microbiomes in lignocellulose degradation has been extensively reported in nature, in mammals [e.g., historically in ruminants (Gordon and Phillips, 1998); and recently more in particular in monkeys (Xu et al., 2015); beavers and mooses (Wong et al., 2016); wallabies (Pope et al., 2010); reindeers (Pope et al., 2012), annelids [e.g., earthworms (Fujii et al., 2012)], and insects [e.g., beetles (Geib et al., 2010; Huang et al., 2012; Sari, 2016)]; cockroaches (Bertino-Grimaldi et al., 2013); and most extensively in termites (Warnecke et al., 2007; Mathew et al., 2013; Brune, 2014; Do et al., 2014)]. Although not representative of the entire gastrointestinal tract, gut microbiomes in giant pandas are generally inferred using microbial communities from stool samples. Such microbial communities have been investigated for their assemblage (Zhu et al., 2011), how they related to dietary shifts (Sims et al., 2007; Williams et al., 2013; Knott et al., 2017) and for their potential cellulose (Zhu et al., 2011), hemicellulose (Zhang et al., 2018) or lignin biodegradation capacity (Fang et al., 2012), as assessed by metagenome analysis. However, no attempt was made so far to cultivate gut microbiomes to mimic giant panda’s feeding. In the present study, we collected the fresh fecal material generated by a young, male panda and used it as microbial inoculum in lab-scale tests. The objective was to put the emphasis on the products of fermentation rather than on its substrates (i.e., the different portions of bamboo). While a relation to the chemical composition of the ‘substrates’ has been made (i.e., the different portions of ingested bamboo), whether panda’s dietary choices also relate to the ‘product’ of bamboo fermentation has been neglected thus far. Here, we focused on the interplay between fermentation of different portions of bamboo of the species *Phyllostachys bisettii* (either the green leaf or the yellow pith), the profile of short-chain organics and biogas produced, the gut microbiome community assemblage (originated from stools either entirely green or yellow) and the microbial metabolism as assessed by metaproteomics.

## Results

### Characterization of Feces and Bamboo

**FIGURE 1:**
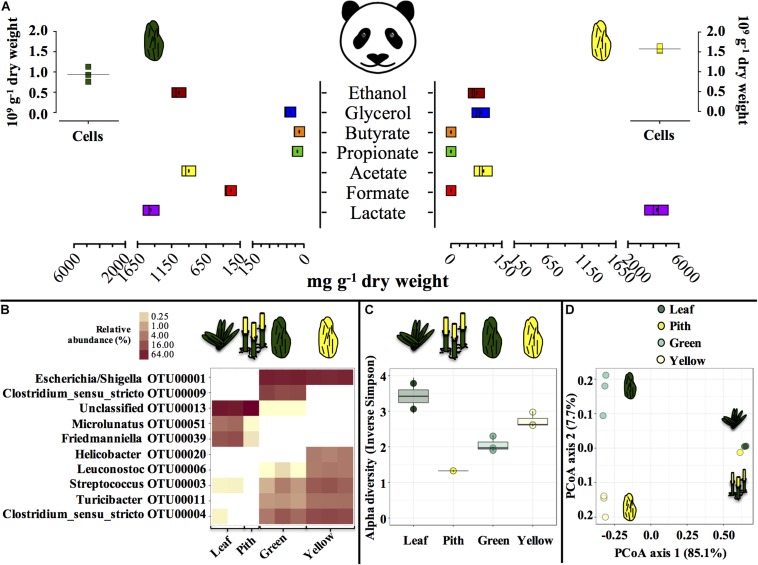
Microbial and biochemical characterization of giant panda fecal matter **(A–D)** and microbial community composition **(B–D)** in *P. bisettii* bamboo fed to the giant panda in the present study. In **(A)**, squares of the same color represent each a single replicate (*n* = 3), with black dots or lines indicating the mean. In **(B–D)**, each single replicate is reported. For plant material (leaf and pith), DNA extraction was conducted on two samples each, however, DNA from one pith sample could not be amplified, therefore the pith is represented by a single sample. Green and yellow dung samples were tested in triplicate (*n* = 3). In **(B)** the top 10 most abundant OTUs across all samples were selected (all data in Supplementary Table S2). In particular, **(C)** reports the Alpha diversity (Hill order 2, Inverse Simpson index) of the microbial community in giant panda fecal matter; circles indicate each single replicate (*n* = 3), whiskers indicate min to max values, and black lines the mean average. In **(D)** the beta diversity analysis by means of principal coordinate analysis (PCoA) of the microbial community in giant panda fecal matter is reported. A 3% dissimilarity threshold was used to define OTUs.

Entirely green or yellow stools from a young, male panda were characterized (Figure 1). Green and yellow stools were typically populated by about 1.0–1.5 × 10^9^ microbial cells g^–1^ dry weight (DW) matter. In green stools, the profile of short-chain organics was a heterogeneous mix of lactate, acetate and ethanol (900 to 1400 mg g^–1^ DW), with minor concentration of formate, contrary to yellow stools where lactate was the only abundant compound (4000 mg g^–1^ DW). *P. bisetti* is a bamboo species commonly offered to pandas in captivity (Edwards et al., 2006). In this study, *P. bisetti* had the typical biochemical composition for the autumn-winter period, i.e., when it was collected (Supplementary Table S1): both leaves and piths have rather high levels of cellulose, hemicellulose and lignin, however, hemicellulose is typically higher in leaves (about 30%) and lignin in piths (about 15%) (Tabet et al., 2004). Predominant genera (Figure 1B) and microbial community assemblage (as assessed by PCoA analysis, Figure 1D) were highly similar in either green leaf or yellow pith of the bamboo species *P. bisetti*, however, they differed remarkably in the green and yellow stools (Supplementary Table S2). This indicates that bamboo passage through the panda’s gut results in enrichment of specific microbial members, organized in a markedly distinct manner as compared to those natively present on the ingested bamboo.

### Effect of Selected Portions of Bamboo and Fecal Microbial Communities on Fermentation

**FIGURE 2:**
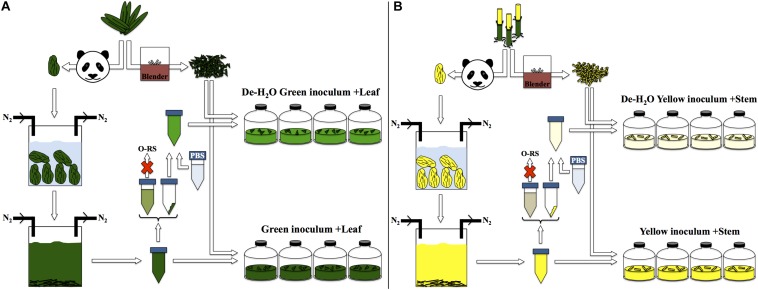
Experimental setup for laboratory-scale fermentation: **(A)** entirely green stools derived from predominant ingestion of *P. bisettii* green leaves were collected and immersed in anaerobic, sterile water. Microbial cells attached to indigested bamboo matter were suspended in the liquid phase along with organics generated in the panda gastrointestinal tract. This mixture was used as inoculum in experiments termed ‘Green inoculum + Leaf’ and supplied with freshly grinded *P. bisettii* bamboo green leaves in multiple independent replicates. In a parallel experiment, such inoculum was centrifuged to separate the organic-rich supernatant (O-RS) from the pellet containing microbial cells and small fragments of indigested bamboo. This dewatering procedure aimed at reducing the impact of the organics derived from the giant panda digestion on the lab-scale experiments. The pellet was resuspended in a phosphate buffer saline (PBS) solution, and used as alternative inoculum supplied with freshly-grinded *P. bisettii* bamboo green leaves in tests termed ‘De-H_2_O green inoculum + Leaf.’ An identical approach was used for yellow stools and yellow pith **(B)**.

The fermentation capacity of different portions of *P. bisetti* by giant panda fecal microbial communities was investigated. A ‘green fermentation line’ was set up using bamboo leaf as substrate, and entirely green fecal samples as starting material to mimic giant panda’s gut microbiomes. Similarly, a ‘yellow fermentation line’ was prepared using the yellow pith, and entirely yellow fecal samples (preparation protocol summarized in Figure 2). The green and yellow bacterial inocula thus prepared carried some soluble organics derived from the actual giant panda’s gut digestion system. Therefore, two other conditions were tested in parallel (one per fermentation line), where the freshly prepared inocula were subjected to centrifugation to remove the soluble organics (Figure 2). The pellet generated by centrifugation contained bacterial biomass along with small fragments of non-digested bamboo; it was re-suspended in a phosphate buffer saline (PBS) solution and provided with fresh bamboo (either leaf or pith). Therefore, accumulation of fermentative products in such de-watered (de-H_2_O) inocula did not depend on soluble organics previously generated in the panda’s gut rather on the fresh, solid bamboo supplied at the beginning of the experiment.

**FIGURE 3:**
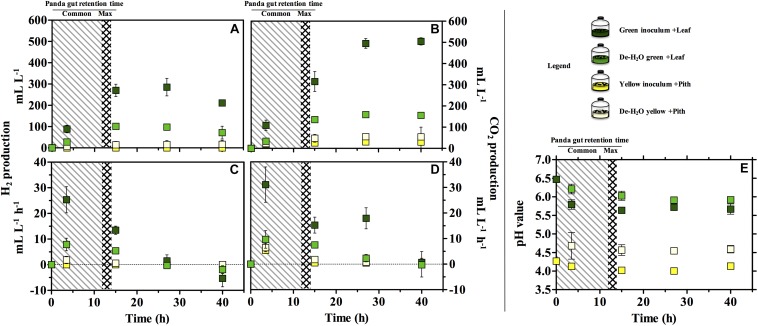
H_2_ **(A,C)**, CO_2_ production **(B,D)** and pH **(E)** in laboratory-scale fermentation tests (*n* = 3) using microbiomes derived from giant panda fecal samples (Figure 2). Common [generally up to 12 h (Dierenfeld et al., 1982; Mainka et al., 1989; Edwards et al., 2006; Senshu et al., 2007)] and maximum [14 h (Schaller et al., 1985)] gut retention times observed in giant pandas are indicated with shaded areas. Keys reported in the graph.

Gas production (either H_2_ or CO_2_) was significantly higher in the green fermentation line as compared to the yellow (log2 fold change [log2_fc_] + 4.61, *p* = 0.007; Figures 3A,B). In particular, H_2_ was produced in very low titers when supplying the yellow pith. In all conditions, gas production rates peaked at 3.5 h of experiment and declined thereafter (Figures 3C,D). Only in the green fermentation line gas production was still substantial after 14 h, which is the longest observed retention time in the giant panda’s gut under normal physiological conditions. The removal of soluble organics tested with de-H_2_O inocula reduced H_2_ and CO_2_ production, with the highly producing green fermentation line the most impacted. H_2_ consumption initiated after 27 h in all cultures that had previously accumulated it (Figure 3C). CH_4_ was never detected in any condition (detection limit 0.01%) demonstrating a purely fermentative metabolism by panda’s fecal microbial communities.

The pH of the two fermentation lines differed markedly (*p* < 0.00001), with the green line around 6.0 and the yellow around 4.5 (Figure 3E). Resuspension in PBS after the dewatering process had little effect on pH in either line despite the initial pH of PBS (which was 7.35).

**FIGURE 4:**
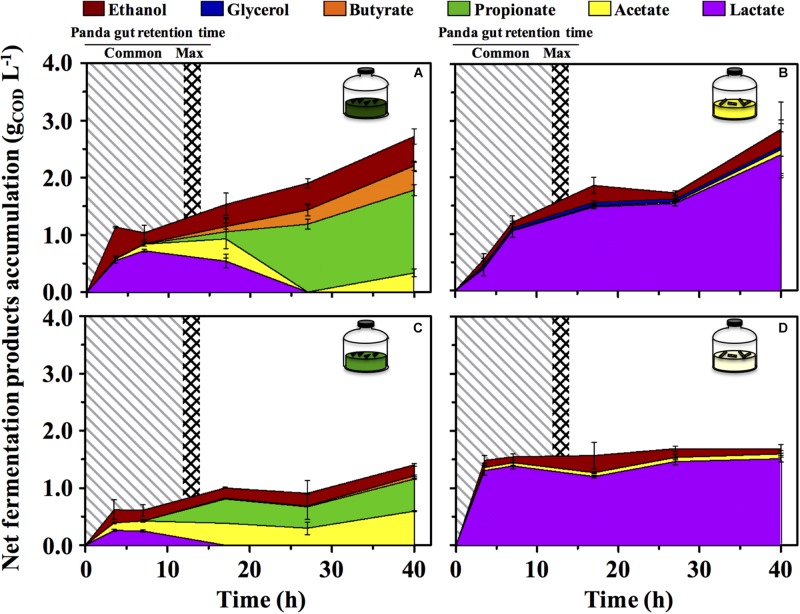
Net fermentative products accumulation in laboratory-scale fermentation tests (*n* = 3) using microbiomes derived from giant panda fecal samples (Figure 2): **(A)** Green inoculum + leaf; **(B)** Yellow inoculum + pith; **(C)** De-H_2_O green inoculum + leaf; **(D)** De-H_2_O yellow inoculum + pith. Common [generally up to 12 h (Dierenfeld et al., 1982; Mainka et al., 1989; Edwards et al., 2006; Senshu et al., 2007)] and maximum [14 h (Schaller et al., 1985)] gut retention times observed in giant pandas are indicated with shaded areas. Keys reported in the graph.

The green fermentation setup resulted in the accumulation of a variety of organics contrary to the yellow, which was dominated by lactic acid (Figure 4). Such fermentation profiles were highly consistent with what detected in the fecal material they originated from, respectively (Figure 1), indicating that our cultivation system was able to reproducibly mimic the giant panda’s gut conditions. When considering the longest retention time observed in panda’s guts [14 h (Schaller et al., 1985)], the net accumulation of fermentative products was slightly affected in the green line (−30% upon dewatering, compare Figures 4A and C) and not affected at all in the yellow line (compare Figures 4B and D). However, fermentation capacity of de-H_2_O inocula remained almost unchanged following 14 h of incubation, contrary to full inocula which reached about 2 times higher concentrations over 40 h (Figure 4). Fermentation profiles were not affected by dewatering in either line. Overall, this indicated that gut microbiomes from both green and yellow stools were consistently capable of fermenting solid bamboo, and could generate short-chain organics at almost equivalent rates within panda’s gut retention times.

In the green line, the fermentative products which accumulated most rapidly (within the first 3.5 h, Figure 4) were lactate and ethanol, along with traces of acetate. In particular, ethanol net production was higher than 1.1 g_COD_ L^–1^ (about 250 mg L^–1^ or about 3%, v:v), which accumulated within only 3.5 h, that is, well within the shortest retention time commonly observed in giant pandas [5 h (Dierenfeld et al., 1982)]. This suggests that giant pandas feeding on bamboo leaves may experience high ethanol levels in their gut, in line with ethanol abundance in green stools (Figure 1).

A time-dependent consumption of lactate (starting after 7 h of incubation) was consistent with the production of acetate, propionate and butyrate (Figures 4A,C). Acetate can derive from conversion of acidogenic products (e.g., ethanol oxidation), propionate from lactate reduction, and butyrate from either glucose (or lactate) oxidation, or from acetate reduction. Furthermore, with prolonged retention times the product type shifts from more oxidized compounds, such as acetate or lactate, toward more reduced compounds (Arslan et al., 2016), as in fact observed here with longer fatty acids accumulated more prominently only after 15–20 h. In stark contrast, lactate was steadily produced in the yellow fermentation from 3.5 to 40 h (Figures 4B,D).

In giant panda’s digestibility trials, total solids (TSs) are reduced 7 to 40% within 13 h (Dierenfeld et al., 1982; Mainka et al., 1989; Sims et al., 2007; Finley et al., 2011). The longer incubation time in the present study (40 h) resulted in higher TS reductions, about 50 and 30% in green and yellow full inocula, respectively (Supplementary Figure S1A), with de-H_2_O inocula showing a TS removal > 60% (Supplementary Figure S1A). The latter confirmed that replacing soluble organics resulting from panda’s digestion with PBS forced gut microbiomes to access solid bamboo. A similar trend was observed with volatile solids (VSs) (Supplementary Figure S1B).

### Microbial Communities

**FIGURE 5:**
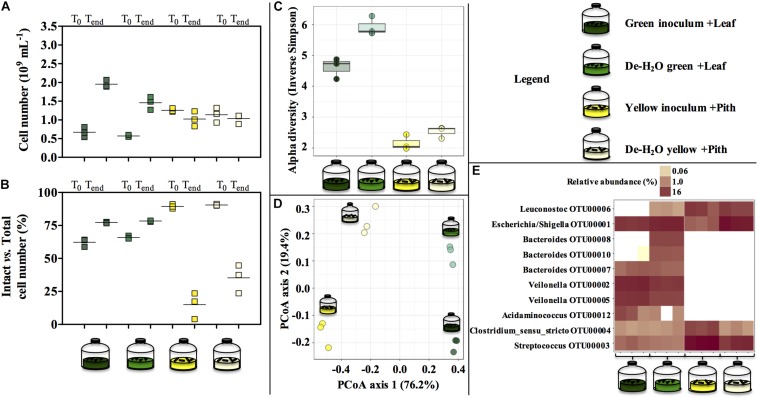
Cell number **(A)**, percentage of intact cells over total cell number **(B)** and microbial community composition **(C–E)** at the end of laboratory-scale incubations (*n* = 3, **A**–**E**) using microbiomes derived from giant panda fecal samples (Figure 2). Total cell number and number intact cells **(A,B)** was determined according to Live-Dead staining and flow cytometry; squares indicate single replicates, and black lines indicate the mean value. Alpha diversity (Hill order 2, Inverse Simpson index) of the microbial community in giant panda fecal matter **(C)**; circles indicate each single replicate, whiskers indicate min to max values, and black lines the mean average. Beta diversity analysis by means of principal coordinate analysis (PCoA) of the microbial community in giant panda fecal matter **(D)**. In **(E)** the top 10 most abundant OTUs across all samples were selected (all data in Supplementary Table S3). A 3% dissimilarity threshold was used to define OTUs. Keys reported in the graph.

Green and yellow fermentation lines had a different cell growth during the incubations. The cell number increased significantly in the green line (from 0.67 to 1.95 × 10^9^ cells mL^–1^ in full inocula log2_fc_ + 1.54, *p* = 0.0003; and from 0.57 to 1.46⋅10^9^ cells mL^–1^ in full de-H_2_O inocula, log2_fc_ + 1.35, *p* = 0.01; Figure 5A), with slightly more intact cells at the end of the incubation (about 75%, Figure 5B). On the contrary, in the yellow line no net increase in cell density was observed while intact cells were strongly reduced (to about 25%, Figure 5B).

The diverse spectrum of short-chain organics in the green fermentation line (Figures 4A,C) was mirrored by a highly diverse group of microbial species (Figure 5C), with *Escherichia*/*Shigella* (OTU00001), *Veilonella* (OTU00002 and OTU00005), *Acidaminococcus* (OTU00012), *Megasphaera* (OTU00023), *Streptococcus* (OTU00003), *Bacteroides* (OTU00008, OTU00010, and OTU00007) and *Clostridium* sensu stricto (OTU00004) above 1% relative abundance (Figure 5E and Supplementary Table S3). Similarly, the accumulation of almost exclusively lactate in yellow inocula (Figures 4B,D) was reflected into communities with low diversity (Figure 5C) where either *Streptococcus* (OTU00003, in Yellow inocula) or *Escherichia/Shigella* (OTU00001, in De-H_2_O yellow inocula) constituted about 60% of the community (Figure 5E and Supplementary Table S3). A Lactobacillales *Leuconostoc*-related strain (OTU00006) was typically associated with yellow rather than green fermentation lines (Figure 5E) (from 0.2 to 14.7%, respectively, log2_fc_ + 6.2, Supplementary Table S3). Microbial community assemblage differed markedly in green and yellow lines, with the removal of soluble organics through dewatering also a factor in shaping communities (Figure 5D). In particular, when forcing communities to feed solely on solid bamboo leaf, the relative abundance of some *Bacteroides* increased (in particular OTU00008 from 0 to 10.4%; and OTU00010 from 0 to 5.3%; Supplementary Table S3) along with *Parabacteroides* (OTU00014, from 0 to 2.0%) and *Morganella* (OTU00019 from 0 to 1.2%, Supplementary Table S3). Concerning the yellow fermentation line, only *Escherischia*/*Shigella* (OTU00001) increased remarkably when forcing communities to feed solely on solid pith (from 4.5 to 52.8%, + 3.6 log2_fc_ Supplementary Table S3).

### Alpha-Amylase From the Giant Panda Is the Predominant Enzyme in Bamboo Fermentation

**FIGURE 6:**
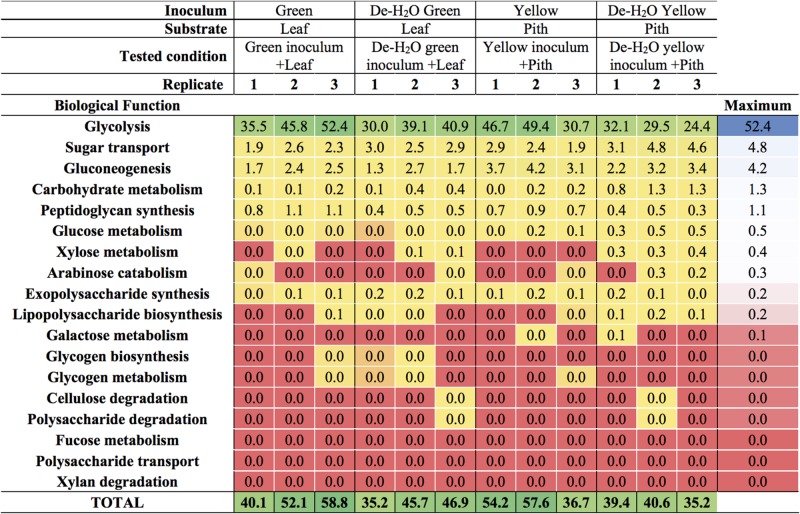
Carbohydrate-related biological functions in laboratory-scale fermentation tests (*n* = 3) using microbiomes derived from giant panda fecal samples (Figure 2). Values represent the percentage of total identified microbial metaproteins.

The predominant microbial pathway in any tested condition was glycolysis (between 30 and 50% of all microbial metaproteins; Figure 6 and Supplementary Table S4), with no difference between culture conditions (*p* > 0.05). Glycolysis is the main metabolic pathway processing glucose, a component of the complex heteropolymer hemicellulose (which also contains xylose, arabinose, mannose, and galactose among others) and the only monomer required to build up the linear polymer cellulose (Perez et al., 2002). Metaproteins related to the functions ‘glucose metabolism,’ ‘xylose metabolism,’ and- to a minor extent- ‘arabinose catabolism’ were also detected in all conditions (up to 0.5% of all microbial metaproteins, Figure 6). These metabolic pathways were particularly relevant in the yellow fermentation line when soluble organics were removed, thus forcing gut microbiomes to degrade the yellow pith (i.e., De-H_2_O yellow inoculum + Pith, Figure 6). Enzymes related to the biological function ‘cellulose degradation’ were < 0.038% of all microbial metaproteins in only two reactors out of 12, and absent in all others. A cellobiose-specific phosphotransferase enzyme (E.C. 2.7.1.205) suggestive of cellulose degradation was detected, however, it was only found in a few replicates of the yellow fermentation and at very low levels (<0.095% of all microbial metaproteins, Supplementary Table S5). Other highly expressed microbial biological functions (>5% of all microbial metaproteins) were virulence, iron storage, transport and protein biosynthesis (Supplementary Table S4; more results discussed in the Supplemental Information). Metaproteins from yeasts were detected, however, apart from an elongation factor (whose relative abundance was between 1.1 up to 3.1%) the sum of all other metaproteins associated with yeasts was not higher than 0.03% in any of the 12 reactors (Supplementary Table S5).

**FIGURE 7:**
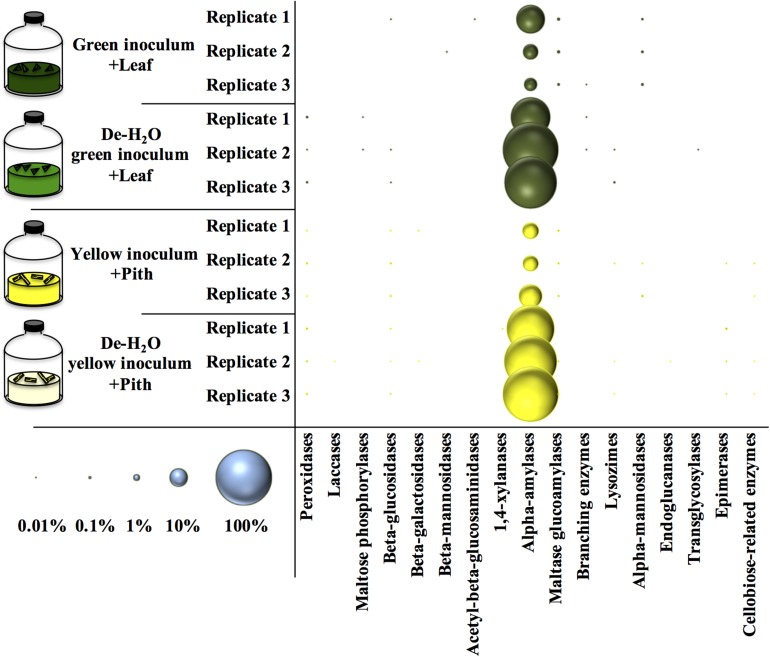
Relative amount of carbohydrate active enzymes (Cazy) at the end of laboratory-scale fermentation tests (*n* = 3) using microbiomes derived from giant panda fecal samples (Figure 2). E.C. numbers and relative abundances for Cazy reported in Supplementary Table S6; list of all metaproteins in Supplementary Table S7. Blue circles (bottom left) indicate the scale of relative abundance of all metaproteins (from 0.01 to 100%). Keys reported in the graph.

An analysis of all the metaproteins was conducted including those not taxonomically related to microbial species. Among the carbohydrate active enzymes (CAZy), alpha-amylases were the most represented metaprotein (Figure 7) and almost exclusively assigned to the giant panda (*A. melanoleuca*) (up to 60% of all identified metaproteins, Supplementary Table S6), with identification of three sequences from Metazoa owing to the high similarity with panda’s alpha-amylases (Supplementary Table S6; all metaproteins in Supplementary Table S7). All identified peptides of alpha-amylase in the giant panda were assigned to the UniProt accession G1L4F3_AILME, although several genes related to alpha-amylase are described in the genome of giant pandas. Loss of material during the dewatering process of the inocula was expected to potentially reduce the amount of water-soluble alpha amylases present in any de-H_2_O reactor. On the contrary, in either green or yellow fermentation line, alpha-amylases from the panda were far more abundant when soluble organics were removed through dewatering (*p* < 0.033, log2_fc_ > + 2.68; Figure 7 and Supplementary Table S6), indicating that panda’s alpha-amylases were attached to their solid substrate. Total metaprotein counts were higher in de-H_2_O reactors at the end of the experiment (+ 0.4 and + 0.7 log2_fc_ in green and yellow line, respectively) excluding a ‘dilution effect’ on relative abundance. The high amount of alpha-amylases in de-H_2_O reactors suggests that they were equally concentrated in both full and de-H_2_O inocula at the onset of the incubation, however, their activity may have decreased in full inocula due to the availability of soluble organics (e.g., Figure 1A).

Many other enzymes related to cellulose, hemicellulose, or lignin degradation were detected (Figure 7), however, their concentration was below 0.1% (Supplementary Table S6). Among these, maltase glucoamylases and alpha-mannosidases from the giant panda were significantly more abundant in the green fermentation line when soluble organics were maintained in the inoculum (*p* < 0.032; Supplementary Table S6). Besides, some uncharacterized proteins of the giant panda were detected. Sequence motifs assigned in UniProt suggest potential protease activity of several hits (Supplementary Table S8). The protein D2HX31_AILME shows a sequence motif related to jacalin-type lectin. Jacalin-related human ZG16p lectin binds oligomeric sugars and is involved in formation of pancreatic zymogen granules as well as in protection against invading pathogen in colon (Kanagawa et al., 2014).

## Discussion

Understanding the feeding behavior of the giant panda, the flagship of endangered species with less than 2000 individuals [in the wild and captivity (Huang et al., 2015)] is a critical part of its conservation. Besides, this offers the opportunity to gain knowledge from a unique microbiome, exposed to a high load of lignocellulosic bamboo within a timespan of hours. In the present study, giant panda gut microbiomes were cultivated for the first time under laboratory conditions. Experiments aimed at revealing how microbial community assemblage, fermentation products and metaproteome profiles were influenced after simulation of giant panda’s selectiveness for different bamboo portions. This was attained through either uniquely ‘green’ or ‘yellow’ lab-scale fermentations, where both bamboo portions (either leaf or pith, respectively) and their correspondent gut microbiomes were used (Figure 2).

Bamboo digestion followed a distinctive pattern strictly related to the portion of bamboo used as substrate: fermentation of green leaves occurred at a close to neutral pH and generated ethanol, lactate and acetate (Figures 3, 4). The yellow pith occurred at more acidic pH and formed lactate almost exclusively (Figures 3, 4). Such fermentation profiles matched those detected in the stools from which the gut microbiomes originated (Figure 1), indicating that the present setup was representative of the actual fermentative patterns occurring in the giant panda’s gut. In particular, in green leaves ethanol rapidly accumulated to strikingly high concentrations (about 3%, v:v).

Irrespective of the fermentation type, glycolysis was the predominant carbohydrate-related microbial biological function assessed by metaproteomics, with microbial metaproteins of glucose and xylose metabolism and- to a lesser extent- arabinose catabolism detected up to 5% (Figure 6). As glucose, xylose and arabinose are found in hemicellulose, with glucose the only monomer required to build up cellulose (Perez et al., 2002), the present data suggests that the panda’s gut microbiomes degraded hemicellulose. Metagenome analysis supports the capacity of gut microbiomes inferred from fecal microbial communities to degrade cellulose (Zhu et al., 2011) and hemicellulose (Zhang et al., 2018), although to a limited degree (Guo et al., 2018). As enzymes related to cellulose digestion were found to very low concentrations or were below detection limits (Supplementary Table S6), our data is in agreement with a limited potential of cellulose degradation in giant panda gut microbiomes.

Xylose and arabinose are pentoses, monosaccharides made of five carbon atoms. In heterofermentation, pentoses are used to produce lactate, CO_2_ and ethanol/acetate mixtures, as opposed to homofermentation where hexoses such as glucose enter glycolysis to generate almost solely lactate (Axelsson and Ahrné, 2000). While showing comparable microbial metaproteomes (Supplementary Table S4), the fermentative product profiles of green leaves typically resembled that of a heterofermentation, as opposed to the yellow pith which was a typical homofermentation to lactate (Figures 1, 4). How these fermentation profiles affect the feeding strategy of giant pandas and relate to its dietary shift to more pith during the spring time should be investigated more accurately with trials involving pandas, although in practice these are highly challenging. Such tests may consider *Bacteroides* (in green leaf) and *Escherichia*/*Shigella* (in yellow pith) as biomarkers indicative of solid lignocellulose degradation, as highlighted by the increase in their abundance upon removal of soluble organics (Supplementary Table S3).

When considering all metaproteins, the most abundant enzyme was the alpha amylase (E.C. 3.2.1.1) from the giant panda itself (Figure 7 and Supplementary Table S6). In de-H_2_O yellow fermentations, where the solid yellow pith was the only carbon source, alpha-amylases constituted up to 60% of all the identified metaproteins in the sample, however, high levels were found in all tested condition (Figure 7). The copy number of alpha-amylase genes is higher in giant panda genomes than in other herbivores, and following the dietary shift from breast milk to bamboo a higher amount of alpha-amylase family genes is detected in metagenomes (Zhang et al., 2018). Since giant panda’s fecal samples had alpha-amylase activities higher than protease or lipase, it was suggested that they could play a role in bamboo digestion (Zheng et al., 2009). The present investigation is the first to report the high abundance of alpha-amylases from the giant panda from actual bamboo fermentation tests. In particular, their high abundance was strictly correlated with the removal of soluble organics in either fermentation line (Figure 7), indicating that they are directly related to solid bamboo digestion. This appears suggestive of a host–microbiome interaction in giant pandas’ gut aimed at achieving lignocellulose degradation. A glucoamylase from the fungus *Termitomyces clypeatus* hydrolized larch wood xylan independently and synergistically with a xylanase, thereby liberating, respectively, glucose or glucose and xylose (Khowala et al., 1992). The role of the alpha amylase from the giant pandas should be investigated further.

To summarize, the digestion of different portions of bamboo resulted in distinctive fermentation profiles. The green leaf was typically heterofermented to ethanol, lactate and acetate, while the yellow pith was homofermented to lactate. Microbial metabolic pathways inferred by metaproteomics suggested that hemicellulose, rather than cellulose or lignin, was the main source of energy. Profiling of fermentation products in stools and lab-scale cultivation of gut microbiomes to assess their actual biocatalytic potential should be implemented in large scale studies to explain the peculiar dietary behavior of giant pandas.

## Materials and Methods

### Inoculum Collection, Preparation, and Cultivation System

The fecal material was collected at the Pairi Daiza zoo (Brugelette, Belgium) and derived from the 6-year old male Xing Hui. No contact with the animal occurred during feces or bamboo collection. Entirely green or yellow stools were collected separately in 1 L airtight containers comprising an AnaeroGen^TM^ bag (Oxoid, Hampshire, United Kingdom) to keep anoxic conditions until processing. About 5 kg of stools were collected for each color. Fresh *P. bisettii* bamboo as offered to Xing Hui was collected the same day (about 5 kg of fresh bamboo).

Stools were processed in the laboratory within 2 h following collection. For each stool, the external layer was removed. Stools containing a mixed color or traces of carrots or apples were discarded and only stools made entirely of either green leaves or yellow pith were selected. These were placed in autoclaved, anaerobic, milliQ water and stirred vigorously for 30 min while N_2_ was sparged to maintain anaerobic conditions. These inocula were termed ‘full inocula,’ as they contained microbes from the giant panda’s gut along with the soluble organics derived from panda’s gut digestion. Part of the full inocula from either green or yellow stools were used to generate a second inoculum termed ‘dewatered’ (de-H_2_O) as follows: full inocula were placed in sterile falcon tubes and centrifuged for 10 min at 14000 rpm (Sorval RC5c PLUS, Beckman, Suarlée, Belgium); the supernatant was discarded and pellets resuspended in an equal volume of phosphate buffer saline solution (PBS), which had a pH of 7.35. The preparation method is summarized in Figure 2.

Green inocula were provided with green leaves of *P. bisettii*, while yellow inocula with yellow pith. The feeding strategy of Xing Hui was simulated when selecting leaves and pith. Leaves attached to thin, apical branches were collected. Leaves were milled into particles of about 1 mm using an electric grinder. The pith was generated by using a file on the yellow portion of the bamboo, which results from peeling off the green external cuticle of stems. The pith was also grinded into particles of about 1 mm. Inocula and bamboo were incubated during 40 h in batch in serum bottles of 120 mL, with 50 mL of either green or yellow inocula provided with 1 g of green leaves or yellow pith, respectively. Reactors were capped using rubber stoppers and sealed with aluminum caps, their headspace was flushed with N_2_ for 15 min to keep anaerobiosis, and they were finally incubated at 37°C (the panda’s body temperature) in a shaking water bath (90 rpm, GLS Aqua 18 Plus, Grant).

### Microbiological Analysis

Cell count and intact/damaged cell count was performed by flow cytometry. SYBR^®^ Green I (10^4^ concentrate in DMSO, Invitrogen) and Propidium Iodide (20 mM in dimethyl sulfoxide [DMSO], LIVE/DEAD BacLight Kit, Invitrogen, Belgium) were used to discriminate cells with intact and damaged cytoplasmic membranes. Flow cytometry was performed using a CyAn^TM^ ADP LX flow cytometer (Dakocytomation, Heverlee, Belgium). MilliQ water was used as the sheath fluid. Data for 20000 events for each sample run was collected.

### Molecular Analysis

DNA extraction for microbial community analysis was conducted on 1 g bamboo material, 1 g of fecal sample, or on the pellet resulting from 2 mL of either full inocula or de-H_2_O reactors. The DNA was extracted with phenol-chloroform and precipitated with ice-cold isopropyl alcohol and 3M sodium acetate. DNA pellets were dried and resuspended in TE buffer and stored at −20°C. DNA quality was assessed using 1% (w:v) agarose gel electrophoresis (Life technologies^TM^, ES), and quantified by a fluorescence assay (QuantiFluor^®^ dsDNA kit; Promega, United States) using a Glomax^®^-Multi + system (Promega). Samples were normalized to 1 ng DNA μL^–1^ and sent to LGC Genomics (DE) for library preparation and sequencing via an Illumina Miseq platform (see Supplementary Information).

### Metaproteomics

Culture samples (30 mL) were centrifuged, pellets resuspended in 2 mL 50 mM Tris/HCl (pH 6.8) and protein extracted with liquid phenol (Heyer et al., 2013). After protein quantification with amido black assay, 25 μg of proteins were loaded into a 12% SDS-PAGE. The SDS-PAGE was conducted after proteins entered approximately 5 mm into the separation gel. The complete protein fraction was digested with trypsin, and peptides were measured by LC-MS/MS using an Elite Hybrid Ion Trap Orbitrap MS with a 120 min gradient. For protein identification, a database search with Mascot (Perkins et al., 1999) was performed, using a false discovery rate of 1% (see Supplementary Information).

### Chemical Analysis

Lignocellulose content in bamboo was measured following the Van Soest method (Van Soest, 1963); TS and VS according Standard Method 2540G (APHA, 1992); and the pH using a pH probe (Herisau, Metrohm, Switzerland). Gas composition was analyzed with a Compact GC (Global Analyser Solutions, Breda, Netherlands), equipped with a Molsieve 5Å pre-column and two channels. Lactic and formic acid were determined with a 930 Compact IC Flex (Metrohm, Switzerland) ion chromatography (IC) system with inline bicarbonate removal (MCS), equipped with a guard column cartridge (Metrosep Dual 4/4.6, Metrhom) and an organic acid column (Metrosep 250/7.8, Metrohm) with a 850 IC conductivity detector. Alcohols including glycerol and ethanol were determined with the same IC equipped with a guard column cartridge (Metrosep Trap 1 100/4.0, Metrohm) and a Metrosep Carb 2 250/4.0 column (Metrohm) with an IC amperometric detector (see Supplementary Information).

### Statistical Analysis

All statistical analyses were performed in the R statistical environment (v3.5.1) (R Core Team, 2015), using functions from the phyloseq (v1.16.2), DESeq2 (v1.22.1) and Phenoflow (v1.1) packages (McMurdie and Holmes, 2013; Love et al., 2014). Alpha diversity was assessed by the Hill diversity numbers, which incorporate both richness and evenness components (Hill, 1973). For beta diversity analysis the taxon abundances were rescaled by calculating their proportions and multiplying them by the minimum sample size present in the data set (McMurdie and Holmes, 2014). Beta diversity was then assessed by principal coordinate analysis (PCoA) of the Bray-Curtis dissimilarity matrix (see Supplementary Information). Results are the mean value of experiments made in 3–4 independent replicates, with error bars indicating standard deviation. Statistical significance was assessed using a non-parametric test (Mann–Whitney test) considering a two-sided distribution with 95% confidence interval.

## Data Availability Statement

The mass spectrometry proteomics data was deposited to the ProteomeXchange Consortium via the PRIDE partner repository with the dataset identifier PXD010872. The data on 16S sequences was deposited to NCBI under the BioProject ID: PRJNA574018.

## Ethics Statement

Samples were collected under the strict supervision of Pairi Daiza zoo personnel. However, no contact with the animal occurred during feces or bamboo collection.

## Author Contributions

AS conceived, designed and performed the experiments, and wrote the manuscript. WK performed the experiments. MC designed and performed the experiments, and co-wrote the manuscript. RH performed the metaproteome analyses and co-wrote the manuscript. RP performed the 16S rRNA analyses and statistical analysis. JS performed the lignocellulose analysis. DB performed the metaproteome data analysis and co-wrote the manuscript. TB, DL, and HZ provided access to the giant panda fecal samples and bamboo. KR funded and supervised the project. All authors reviewed the manuscript.

## Conflict of Interest

TB is employed by Pairi Daiza Foundation. The remaining authors declare that the research was conducted in the absence of any commercial or financial relationships that could be construed as a potential conflict of interest.

## References

[B1] APHA (1992). *Standard Methods for the Examination of Water and Wastewater*, 18th Edn, Washington DC: American Public Health Association.

[B2] Arslan D. Steinbusch K. J. J. Diels L. Hamelers H. V. M. Strik D. P. B. T. B. Buisman C. J. N. (2016). Selective short-chain carboxylates production: A review of control mechanisms to direct mixed culture fermentations. *Crit. Rev. Environ. Sci. Technol.* 46 592–634. 10.1080/10643389.2016.1145959

[B3] Axelsson L. Ahrné S. (2000). “Lactic acid bacteria,” in *Applied Microbial Systematics*, eds Priest F. G. Goodfellow M. (Berlin: Kluwer Academic Publishers), 367–388.

[B4] Bertino-Grimaldi D. Medeiros M. N. Vieira R. P. Cardoso A. M. Turque A. S. Silveira C. B. (2013). Bacterial community composition shifts in the gut of *Periplaneta americana* fed on different lignocellulosic materials. *Springerplus* 2:609. 10.1186/2193-1801-2-609 24324923 PMC3855920

[B5] Brune A. (2014). Symbiotic digestion of lignocellulose in termite guts. *Nat. Rev. Microbiol.* 12 168–180. 10.1038/nrmicro3182 24487819

[B6] Dierenfeld E. S. (1997). “Chemical composition of bamboo in relation to giant panda nutrition,” in *The Bamboos*, ed. Chapman G. P. (London: Academic press), 205–211.

[B7] Dierenfeld E. S. Hintz H. F. Robertson J. B. Van Soest P. J. Oftedalt A. T. (1982). Utilization of bamboo by the giant panda. *J. Nutr.* 112 636–641. 10.1093/jn/112.4.636 6279804

[B8] Do T. H. Nguyen T. T. Nguyen T. N. Le Q. G. Nguyen C. Kimura K. (2014). Mining biomass-degrading genes through Illumina-based de novo sequencing and metagenomic analysis of free-living bacteria in the gut of the lower termite *Coptotermes gestroi* harvested in Vietnam. *J. Biosci. Bioeng.* 118 665–671. 10.1016/j.jbiosc.2014.05.010 24928651

[B9] Edwards M. S. Zhang G. Wei R. Liu X. (2006). “Nutrition and dietary husbandry,” in *Giant Pandas: Biology, Veterinary Medicine and Management*, eds Wildt D. E. Zhang A. Zhang H. Janssen D. L. Ellis S. (Cambridge: Cambridge University Press), 101–158. 10.1017/cbo9780511542244.007

[B10] Fang W. Fang Z. Zhou P. Chang F. Hong Y. Zhang X. (2012). Evidence for lignin oxidation by the giant panda fecal microbiome. *PLoS One* 7:e50312. 10.1371/journal.pone.0050312 23209704 PMC3508987

[B11] Finley T. G. Sikes R. S. Parsons J. L. Rude B. J. Bissell H. A. Ouellette J. R. (2011). Energy digestibility of giant pandas on bamboo-only and on supplemented diets. *Zoo. Biol.* 30 121–133. 10.1002/zoo.20340 20814990

[B12] Fujii K. Ikeda K. Yoshida S. (2012). Isolation and characterization of aerobic microorganisms with cellulolytic activity in the gut of endogeic earthworms. *Int. Microbiol.* 15 121–130. 10.2436/20.1501.01.165 23847816

[B13] Geib S. M. Tien M. Hoover K. (2010). Identification of proteins involved in lignocellulose degradation using in gel zymogram analysis combined with mass spectroscopy-based peptide analysis of gut proteins from larval Asian longhorned beetles, *Anoplophora glabripennis*. *Insect Sci.* 17 253–264. 10.1111/j.1744-7917.2010.01323.x

[B14] Gittleman J. L. (1994). Are the pandas successful specialists or evolutionary failures? *Bioscience* 44 456–464. 10.2307/1312297

[B15] Gordon G. L. Phillips M. W. (1998). The role of anaerobic gut fungi in ruminants. *Nutr. Res. Rev.* 11 133–168. 10.1079/NRR19980009 19087463

[B16] Guo W. Mishra S. Zhao J. Tang J. Zeng B. Kong F. (2018). Metagenomic study suggests that the gut microbiota of the giant panda (ailuropoda melanoleuca) may not be specialized for fiber fermentation. *Front. Microbiol.* 9:229. 10.3389/fmicb.2018.00229 29503636 PMC5820910

[B17] Hansen R. L. Carr M. M. Apanavicius C. J. Jiang P. Bissell H. A. Gocinski B. L. (2010). Seasonal shifts in giant panda feeding behavior: relationships to bamboo plant part consumption. *Zoo. Biol.* 29 470–483. 10.1002/zoo.20280 19862794

[B18] Heyer R. Kohrs F. Benndorf D. Rapp E. Kausmann R. Heiermann M. (2013). Metaproteome analysis of the microbial communities in agricultural biogas plants. *New Biotechnol.* 30 614–622. 10.1016/j.nbt.2013.01.002 23369865

[B19] Hill M. O. (1973). Diversity and evenness: a unifying notation and its consequences. *Ecology* 54 427–432. 10.2307/1934352

[B20] Huang J. Li Y. Z. Du L. M. Yang B. Shen F. J. Zhang H. M. (2015). Genome-wide survey and analysis of microsatellites in giant panda (Ailuropoda melanoleuca), with a focus on the applications of a novel microsatellite marker system. *BMC Genom.* 16:61. 10.1186/s12864-015-1268-z 25888121 PMC4335702

[B21] Huang S. Sheng P. Zhang H. (2012). Isolation and identification of cellulolytic bacteria from the gut of *Holotrichia parallela* larvae (Coleoptera: Scarabaeidae). *Int. J. Mol. Sci.* 13 2563–2577. 10.3390/ijms13032563 22489111 PMC3317674

[B22] Jin C. Ciochon R. L. Dong W. Hunt R. M. Liu J. Jaeger M. (2007). The first skull of the earliest giant panda. *Proc. Natl. Acad. Sci. U.S.A.* 104 10932–10937. 10.1073/pnas.0704198104 17578912 PMC1904166

[B23] Kanagawa M. Liu Y. Hanashima S. Ikeda A. Chai W. Nakano Y. (2014). Structural basis for multiple sugar recognition of Jacalin-related human ZG16p lectin. *J. Biol. Chem.* 289 16954–16965. 10.1074/jbc.M113.539114 24790092 PMC4059138

[B24] Khowala S. Ghosh A. K. Sengupta S. (1992). Saccharification of xylan by an amyloglucosidase of termitomycesclypeatusand synergism in the presence of xylanase. *Appl. Microbiol. Biotechnol.* 37 287–292. 10.1007/bf00210979

[B25] Knott K. K. Christian A. L. Falcone J. F. Vance C. K. Bauer L. L. Fahey G. C. (2017). Phenological changes in bamboo carbohydrates explain the preference for culm over leaves by giant pandas (Ailuropoda melanoleuca) during spring. *PLoS One* 12:e0177582. 10.1371/journal.pone.0177582 28614359 PMC5470666

[B26] Li R. Fan W. Tian G. Zhu H. He L. Cai J. (2010). The sequence and de novo assembly of the giant panda genome. *Nature* 463 311–317. 10.1038/nature08696 20010809 PMC3951497

[B27] Love M. I. Huber W. Anders S. (2014). Moderated estimation of fold change and dispersion for RNA-seq data with DESeq2. *Genome Biol.* 15:550. 10.1186/s13059-014-0550-8 25516281 PMC4302049

[B28] Mainka S. A. Guanlu Z. Mao L. (1989). Utilization of a bamboo, sugar cane, and gruel diet by two juvenile giant pandas (*Ailuropoda melanoleuca*). *J. Zoo Wildlife Med.* 20 39–44.

[B29] Mathew G. M. Mathew D. C. Lo S. C. Alexios G. M. Yang J. C. Sashikumar J. M. (2013). Synergistic collaboration of gut symbionts in *Odontotermes formosanus* for lignocellulosic degradation and bio-hydrogen production. *Bioresour. Technol.* 145 337–344. 10.1016/j.biortech.2012.12.055 23298769

[B30] McMurdie P. J. Holmes S. (2013). phyloseq: an R package for reproducible interactive analysis and graphics of microbiome census data. *PLoS One* 8:e61217. 10.1371/journal.pone.0061217 23630581 PMC3632530

[B31] McMurdie P. J. Holmes S. (2014). Waste not, want not: why rarefying microbiome data is inadmissible. *PLoS Comput. Biol.* 10:e1003531. 10.1371/journal.pcbi.1003531 24699258 PMC3974642

[B32] Nie Y. Speakman J. R. Wu Q. Zhang C. Hu Y. Xia M. (2015). Animal Physiology. Exceptionally low daily energy expenditure in the bamboo-eating giant panda. *Science* 349 171–174. 10.1126/science.aab2413 26160943

[B33] Nie Y. Wei F. Zhou W. Hu Y. Senior A. M. Wu Q. (2019). Giant pandas are macronutritional carnivores. *Curr. Biol.* 29 1677–1682. 10.1016/j.cub.2019.03.067 31056385

[B34] Perez J. Munoz-Dorado J. de la Rubia T. Martinez J. (2002). Biodegradation and biological treatments of cellulose, hemicellulose and lignin: an overview. *Int. Microbiol.* 5 53–63. 10.1007/s10123-002-0062-3 12180781

[B35] Perkins D. N. Pappin D. J. C. Creasy D. M. Cottrell J. S. (1999). Probability-based protein identification by searching sequence databases using mass spectrometry data. *Electrophoresis* 20 3551–3567. 10.1002/(sici)1522-2683(19991201)20:18<3551::aid-elps3551>3.0.co;2-2 10612281

[B36] Pope P. B. Denman S. E. Jones M. Tringe S. G. Barry K. Malfatti S. A. (2010). Adaptation to herbivory by the tammar wallaby includes bacterial and glycoside hydrolase profiles different from other herbivores. *Proc. Natl. Acad. Sci. U.S.A.* 107 14793–14798. 10.1073/pnas.1005297107 20668243 PMC2930436

[B37] Pope P. B. Mackenzie A. K. Gregor I. Smith W. Sundset M. A. McHardy A. C. (2012). Metagenomics of the svalbard reindeer rumen microbiome reveals abundance of polysaccharide utilization loci. *PLoS One* 7:e38571. 10.1371/journal.pone.0038571 22701672 PMC3368933

[B38] R Core Team (2015). *A Language And Environment for Statistical Computing. R Foundation For Statistical Computing.* Vienna: R Core Team.

[B39] Rybiski Tarou L. Williams J. Powell D. M. Tabet R. Allen M. (2005). Behavioral preferences for bamboo in a pair of captive giant pandas (Ailuropoda melanoleuca). *Zoo Biol.* 24 177–183. 10.1002/zoo.20038

[B40] Sari S. L. A. (2016). Cellulolytic and hemicellulolytic bacteria from the gut of Oryctes rhinoceros larvae. *Biodiv. J. Biol. Diver.* 17 78–83. 10.13057/biodiv/d170111

[B41] Schaller G. Hu J. Pan W. Zhu J. (1985). *The Giant Pands OF Wolong.* Chicago, IL: University of Chicago Press.

[B42] Senshu T. Miyata K. Ohya A. Mikogai J. Morita M. Nakao T. (2014). Procedure and mechanisms of bamboo cell wall digestion in the giant panda, *Ailuropoda melanoleuca*. *Mamm. Study* 39 219–228. 10.3106/041.039.0405

[B43] Senshu T. Ohya A. Ide K. Mikogai J. Morita M. Nakao T. (2007). Studies on the digestion in the giant panda, *Ailuropoda melanoleuca*, fed feedstuffs including bamboo. *Mamm. Study* 32 139–149. 10.3106/1348-6160(2007)32[139:sotdit]2.0.co;2

[B44] Sims J. A. Parsons J. L. Bissell H. A. Sikes R. S. Ouellette J. R. Rude B. J. (2007). Determination of bamboo-diet digestibility and fecal output by giant pandas. *Ursus* 18 38–45. 10.2192/1537-6176(2007)18[38:dobdaf]2.0.co;2

[B45] Tabet R. B. Oftedal O. T. Allen M. E. (2004). “Seasonal differences in composition of bamboo fed to giant pandas (Ailuropoda melanoleuca) at the National Zoo,” in *Proceedings of the Fifth Comparative Nutrition Society Symposium*, Hickory Corners, MI.

[B46] Taylor A. H. Zisheng Q. (1987). Culm dynamics and dry matter production of bamboos in the wolong and tangjiahe giant panda reserves, sichuan, China. *J. Appl. Ecol.* 24 419–433. 10.2307/2403884

[B47] Van Soest P. J. (1963). Use of detergents in the analysis of fibrous feeds. II. A rapid method for the determination of fiber and lignin. *J. Sci.* 45 829–835. 10.1093/jaoac/46.5.829

[B48] Warnecke F. Luginbuhl P. Ivanova N. Ghassemian M. Richardson T. H. Stege J. T. (2007). Metagenomic and functional analysis of hindgut microbiota of a wood-feeding higher termite. *Nature* 450 560–565. 10.1038/nature06269 18033299

[B49] Wildt D. E. Zhang A. Zhang H. Janssen D. L. Ellis S. (2006). *Giant Pandas - Biology, Veterinary Medicine and Management.* Cambridge: Cambridge University Press.

[B50] Williams C. L. Willard S. Kouba A. Sparks D. Holmes W. Falcone J. (2013). Dietary shifts affect the gastrointestinal microflora of the giant panda (Ailuropoda melanoleuca). *J. Anim. Physiol. Anim. Nutr.* 97 577–585. 10.1111/j.1439-0396.2012.01299.x 22524500

[B51] Wong M. T. Wang W. Lacourt M. Couturier M. Edwards E. A. Master E. R. (2016). Substrate-driven convergence of the microbial community in lignocellulose-amended enrichments of gut microflora from the canadian beaver (*Castor canadensis*) and North American Moose (*Alces americanus*). *Front. Microbiol.* 7:961. 10.3389/fmicb.2016.00961 27446004 PMC4914502

[B52] Xu B. Xu W. Li J. Dai L. Xiong C. Tang X. (2015). Metagenomic analysis of the *Rhinopithecus bieti* fecal microbiome reveals a broad diversity of bacterial and glycoside hydrolase profiles related to lignocellulose degradation. *BMC Genom.* 16:174. 10.1186/s12864-015-1378-7 25887697 PMC4369366

[B53] Zhang W. Liu W. Hou R. Zhang L. Schmitz-Esser S. Sun H. (2018). Age-associated microbiome shows the giant panda lives on hemicelluloses, not on cellulose. *ISME J.* 12 1319–1328. 10.1038/s41396-018-0051-y 29391488 PMC5931968

[B54] Zhao H. Yang J. R. Xu H. Zhang J. (2010). Pseudogenization of the umami taste receptor gene Tas1r1 in the giant panda coincided with its dietary switch to bamboo. *Mol. Biol. Evol.* 27 2669–2673. 10.1093/molbev/msq153 20573776 PMC3108379

[B55] Zheng Y. Fei L. Li F. Niu L. Zhang Z. (2009). Analysis of the digestive enzyme activities in the digestive tract of giant pandas. *Sichuan J. Zool.* 28 397–400.

[B56] Zhu L. Wu Q. Dai J. Zhang S. Wei F. (2011). Evidence of cellulose metabolism by the giant panda gut microbiome. *Proc. Natl. Acad. Sci. U.S.A.* 108 17714–17719. 10.1073/pnas.1017956108 22006317 PMC3203778

