# Supplementary material for: Substrate-Dependent Fermentation of Bamboo in Giant Panda Gut Microbiomes: Leaf Primarily to Ethanol and Pith to Lactate
Source: Front Microbiol. 2020 Mar 31;11:530. doi: 10.3389/fmicb.2020.00530 (PMC7145396; doi:10.3389/fmicb.2020.00530)
Supplement: Supplementary file 1 [file Data_Sheet_1.docx]

**Supplementary Information**

Substrate-dependent fermentation of bamboo in giant panda gut microbiomes: leaf primarily to ethanol and pith to lactate

Alberto Scoma^1,2,3*^, Way Cern Khor^1†^, Marta Coma^1^, Robert Heyer^4^, Ruben Props^1^, Jonas Schoelynck^5^, Tim Bouts^6^, Dirk Benndorf^4,7^, Desheng Li^8^, Hemin Zhang^8^, Korneel Rabaey^1^

^1^ Center for Microbial Ecology & Technology (CMET), University of Ghent, Coupure Links 653, 9000 Ghent, Belgium

^2^ Department of Bioscience, Microbiology Section, Aarhus University, Ny Munkegade 116, 8000 Aarhus C, Denmark

^3^ Department of Engineering, Biological and Chemical Engineering (BCE), Aarhus University, Hangøvej 2, 8200 Aarhus N, Denmark

^4^ Otto von Guericke University of Magdeburg, Bioprocess Engineering, Universitätsplatz 2, 39106 Magdeburg, Germany

^5^ University of Antwerp, Universiteitsplein 1, 2610 Wilrijk, Belgium

^6^ Pairi Daiza Foundation, Domaine de Cambron, 7940 Brugelette, Belgium

^7^ Max Planck Institute for Dynamics of Complex Technical Systems, Bioprocess Engineering, Sandtorstraße 1, 39106 Magdeburg, Germany

^8^ China Conservation and Research Centre for Giant Panda (CCRCGP), DuJiangYan City, SiChuan Province, China

* Corresponding author:

Prof. Alberto Scoma

Department of Engineering,

Biological and Chemical Engineering (BCE),

Aarhus University,

Hangøvej 2, 8000, Aarhus C, Denmark

Email: [as@eng.au.dk](mailto:as@eng.au.dk)

Phone: 0045 8715 6549

^†^ This study is dedicated to the memory of Way Cern Khor, a hard-working, passionate young scientist and a dear friend

**Supplemental Results & Discussion**

*Microbial communities on plant material, and in dung and lab-scale incubation samples*

Fresh *P. bisettii* bamboo as fed to the giant panda tested in this study was collected, and the microbial community composition present on either the green leaf or the yellow pith, which were later supplied to gut microbiomes tested in the laboratory, was assessed. DNA extraction and amplification were conducted in triplicate for dung samples and lab-scale incubations, and in duplicate on plant material, however, one sample of the pith could not be amplified (Fig. 1B). Rarefaction curves for 16S rRNA gene raw data used for differential gene expression (DESeq) analysis and for beta-diversity of all samples (plant material, dung samples, and lab-scale incubations of gut microbiomes) are presented in Fig. S2 and Fig. S3, respectively.

A total number of 305 OTUs were found on plant material. OTUs per samples were between 59 and 165, with a number of reads per sample between 1625 and 1976 (Table S2). The most abundant OTU was an unclassified microorganism (OTU00013) in both leaf and pith. OTU00013 constituted 52.8% (mean average, n=2) of the overall microbial community on the leaf, while it made up to 86.9% in the only pith sample which could be amplified (Table S2).

In dung samples, between 30725 and 11374 reads per sample were obtained, with a number of OTUs per sample between 69 and 173. At the end of lab-scale incubations, between 8587 and 36688 reads per sample were obtained, with a number of OTUs per sample between 42 and 192. Overall, the six dung samples analyzed had a total of 564 OTUs, while the 12 lab-scale incubations tested had a total of 1167 OTUs. These values are at the medium to high end of total number of OTUs obtained in giant panda gut microbiome studies so far (118 in (Williams et al., 2016); 235 in (Li et al., 2015); 781 in (Xue et al., 2015); 1161 OTUs in (Zhang et al., 2018)). As opposed to what was detected on the different bamboo portions, in dung samples and at the end of lab-scale incubations the number of reads of the OTU00013 was negligible and did not account for more than 0.1% (Table S2 and Table S3). Dung samples were instead predominated by OTU00001 (closest relative *Escherichia*/*Shigella*) which was 65.9 and 55.9% in green and yellow dung, respectively (mean average, n=3; Table S2). Besides, two OTUs whose closest relative was a Clostridium_sensu_stricto were also abundant in either dung sample (namely, OTU00009, 21.1% in green dung; and OTU00004, 7.7 and 18.9% in green and yellow dung, respectively). Finally, the yellow dung contained a relatively high abundance of OTU00003 (11%, closest relative *Streptococcus*).

*Metaproteomes*

Microbial metaproteins were most largely related to Glycosolysis (38% on average, Table S4), which was statistically comparable among all samples (p> 0.05). Other biological functions were impacted by the experimental set up, most notably the dewatering process that removed the organic-rich supernatant from dung samples to generate either full or de-H_2_O inocula (Fig. 2). When comparing full *vs.* de-H_2_O inocula (in either green or yellow fermentation line) the biological functions Transport and Ion transport were significantly upregulated in the absence of soluble organics (log2 fold change [log2_fc_] between +0.77 and +1.45, *p* <0.025; Table S4). In the absence of abundant nutrients as in full inocula, de-H_2_O gut microbiomes were most active in taking up nutrients from the outer environment as soon as they became available during bamboo degradation. This also enhanced Protein biosynthesis in de-H_2_O gut microbiomes, owing to the absence of readily available amino acids in the medium (log2_fc_ +0.85 and +0.90 in green and yellow tests, respectively, *p* <0.04; Table S4). Peptidoglycan synthesis, cell wall biogenesis/degradation and cell shape were also enhanced in full inocula vs. de-H_2_O inocula (in either the green or yellow line; log2_fc_ between +0.96 and +1.06, *p* <0.02; Table S4). A high level of enzymes related to these functions may result from 1) microbial cells requiring more cell wall components for either growth or cell wall repair; and/or 2) enzymes linked to the degradation of the peptidoglycans present in the organic-rich supernatant removed by dewatering. However, dewatering had no effect on either cell growth or intact/damaged cell ratios (Fig. 5A,B), as the green fermentation line was always subjected to high growth rates and low cell damage, whereas the yellow fermentation line always experienced a slight reduction in cell number and high levels of cell damage. This suggests that part of the organic matter in the organic-rich supernatant incubated with full inocula may have been peptidoglycans.

**Figure S1**. Total solids (**A**) and volatile solids (**B**) at the onset (T_0_) and end (T_end_) of laboratory-scale fermentation tests using microbiomes derived from giant panda gut samples, namely (left to right, in either **A** or **B**): Green inoculum +leaf; De-H_2_O green inoculum +leaf; Yellow inoculum +pith; De-H_2_O yellow inoculum +pith (see Fig. 2 for the complete experimental set up). Squares indicate each single replicate (n=3), and black lines the mean average.

**Figure S2**. Rarefaction curves for 16S rRNA gene raw data used for differential gene expression (DESeq) analysis, taking into account differences in library size.

**
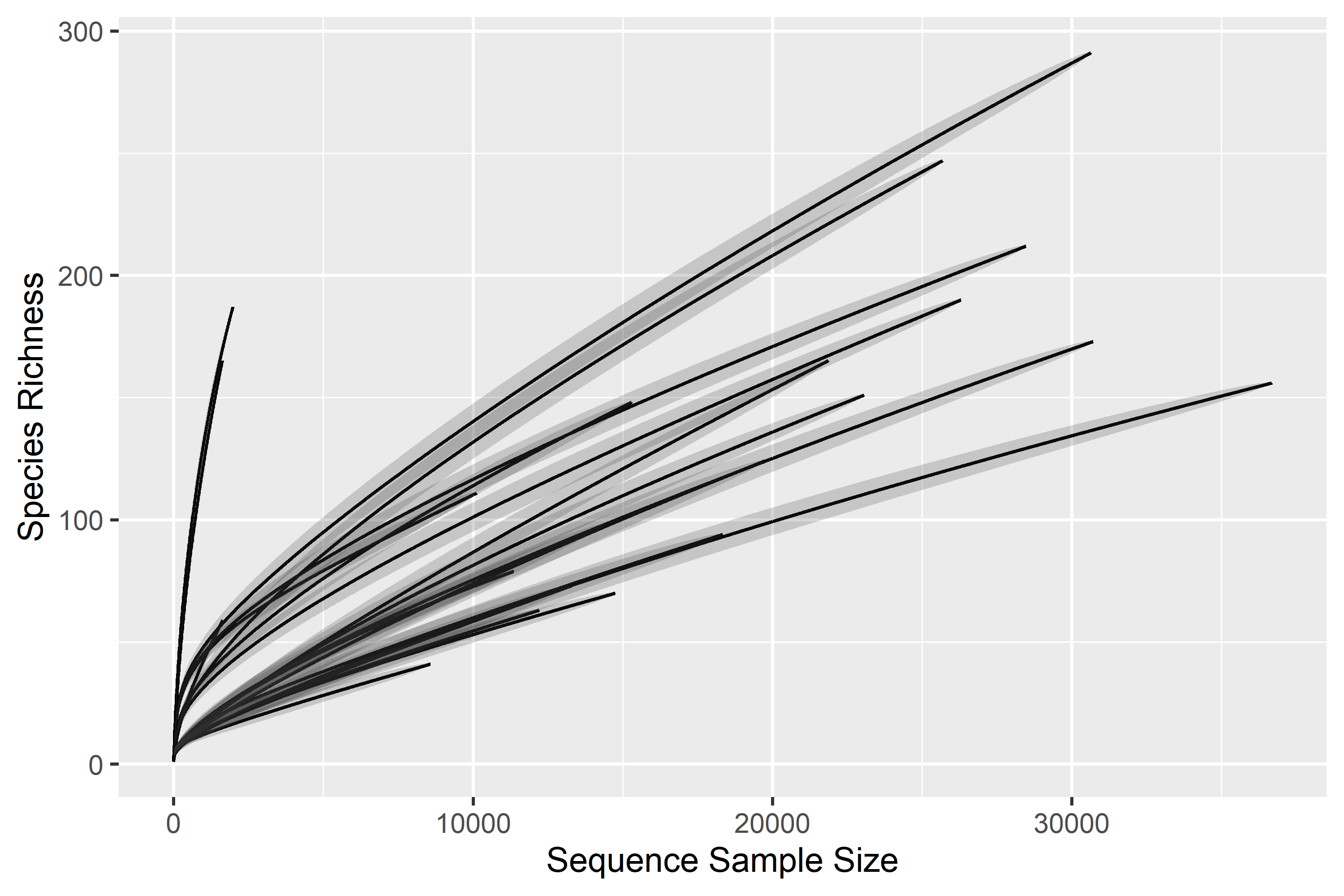
**

**Figure S3**. Rarefaction curves for 16S rRNA gene data re-scaled to 8481 reads for beta-diversity comparisons, accounting for differences in sample size. Nearly all samples flatten out at this number of reads.

**
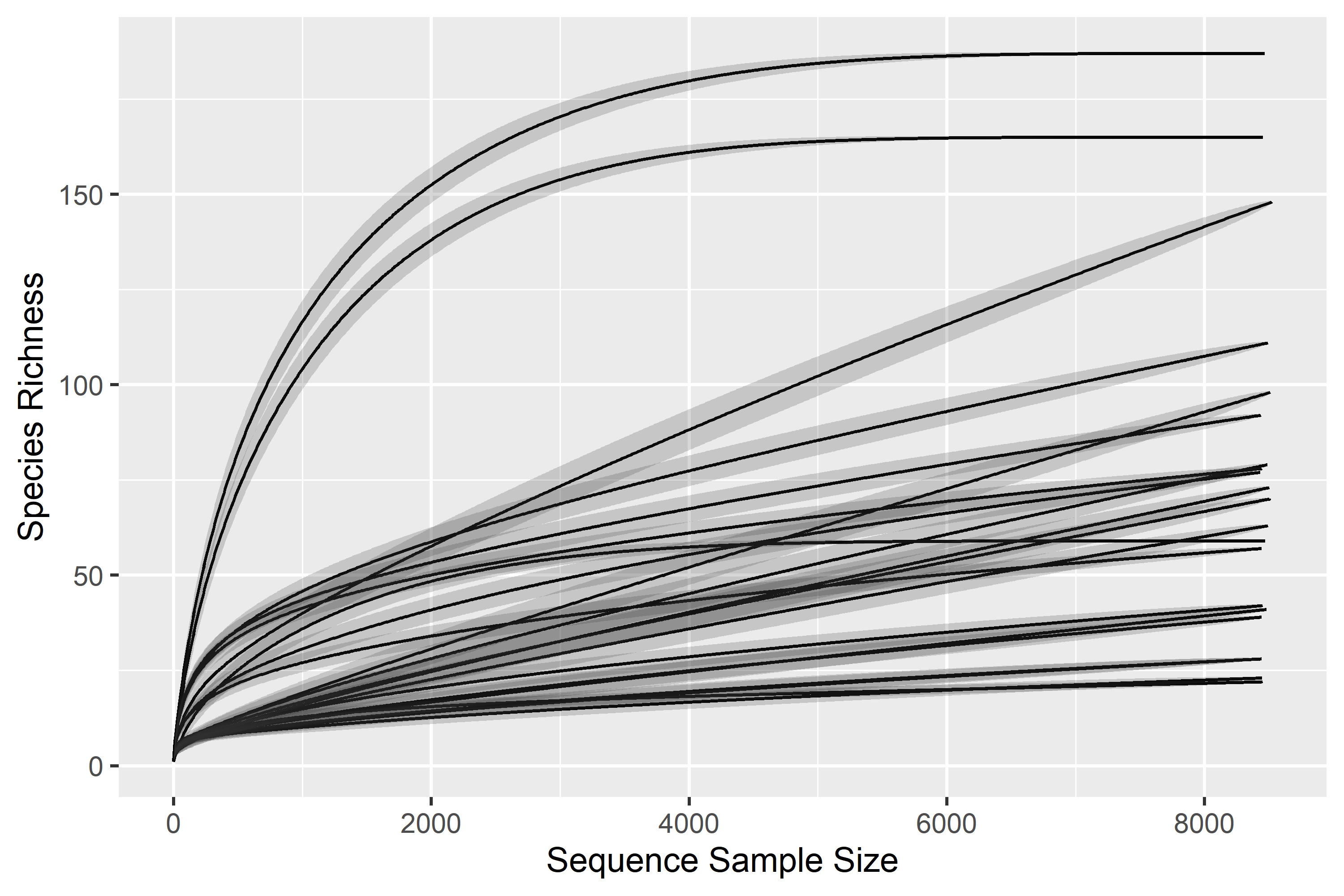
**

**Table S1**. Lignocellulose content in *Phyllostachys bisetti* green leaves and yellow pith used as feed in the present study as compared to the literature (Tabet et al., 2004). Colors in cells are used as a concentration gradient, with dark green indicating high and red indicating low values.

**Table S2**: Total number of reads and relative abundance of 16S rRNA sequences in bamboo plant material fed to the giant panda and in faecal material derived from giant panda (see Figure 2 for the complete experimental set up).

**Table S3**: Relative abundance of 16S rRNA sequences in laboratory-scale fermentation tests using microbiomes derived from giant panda gut samples (see Figure 2 for the complete experimental set up).

**Table S4**. Expression of microbial metaproteins related to biological functions (UniProtBB keyword) in laboratory-scale fermentation tests using microbiomes derived from giant panda gut samples (see Figure 2 for the complete experimental set up).

**Table S5**. Expression of metaproteins (UniProtBB) related to yeasts in laboratory-scale fermentation tests using microbiomes derived from giant panda gut samples (see Figure 2 for the complete experimental set up).

**Table S6**. Expression of metaproteins related to carbohydrate active enzymes (www.cazypedia.org) in laboratory-scale fermentation tests using microbiomes derived from giant panda gut samples (see Figure 2 for the complete experimental set up).

**Table S7**. Expression of metaproteins (UniProtBB) in laboratory-scale fermentation tests using microbiomes derived from giant panda gut samples (see Figure 2 for the complete experimental set up).

**Table S8**. Selected list of uncharacterized proteins whose sequence motif is suggestive of protease activity (UniProt keyword). Complete list of metaproteins in Table S6

**Materials and Methods**

**Microbial cultivation system**

Stools were processed in the laboratory within 2 h following collection. For each stool, the external layer was removed, and the internal part checked for its content. Stools containing a mixed color or traces of carrots or apples were discarded. Only stools made entirely of either green leaves or yellow pith were selected. Selected stools were placed in autoclaved, anaerobic, milliQ water and stirred vigorously for 30 min while N_2_ was sparged to maintain anaerobic conditions. To prepare the green inoculum, the final amount of wet green stools was 1509 g, to which 1536 mL of anaerobic, autoclaved milliQ water was added. For the yellow inoculum, these were 973 g and 882 mL, respectively. These inocula were termed ‘full inocula’, as they contained microbes from the giant panda’s gut along with the soluble organics derived from panda’s gut digestion. Part of the full inocula from either green or yellow stools were used to generate a second inoculum termed ‘dewatered’ (de-H_2_O) as follows: full inocula were placed in sterile falcon tubes and centrifuged for 10 min at 14000 rpm (Sorval RC5c PLUS, Beckman, Suarlée, Belgium); the supernatant was discarded and pellets resuspended in an equal volume of phosphate buffer saline solution (PBS), which had a pH of 7.35. The preparation method is summarized in Fig. 2.

**Microbial cultivation procedures**

Green inocula were provided with green leaves of *P. bisettii*, while yellow inocula with yellow pith. The feeding strategy of Xing Hui was simulated when selecting leaves and pith. Leaves attached to thin, apical branches were collected. Leaves were milled into particles of about 1 mm using an electric grinder. The pith was generated by using a file on the yellow portion of the bamboo, which results from peeling off the green external cuticle of stems. The pith was also grinded into particles of about 1 mm.

Inocula and bamboo were incubated during 40 h in batch in serum bottles of 120 mL, with 50 mL of either green or yellow inocula provided with 1 g of green leaves or yellow pith, respectively. Reactors were capped using rubber stoppers and sealed with aluminum caps, their headspace was flushed with N_2_ for 15 min to keep anaerobiosis, and they were finally incubated at 37 °C (the panda’s body temperature) in a shaking water bath (90 rpm, GLS Aqua 18 Plus, Grant).

A sample at time zero was taken from each reactor before closing the serum bottles. Afterwards, liquid and/or gas samples were taken at 3.5, 7, 17, 27 and 40 h. At each sampling point the volume of gas produced within the bottle was measured with a sterile syringe and analyzed for its composition. 1.5 mL of liquid sample (max. 10% reactor volume for sampling) was collected with a syringe after gas decompression. To prevent the buildup of negative pressure in the reactors, an equal amount of N_2_ was inserted prior to liquid sample collection. pH was measured immediately upon sampling, and the sample filtered at 0.45 μm and kept at -20 °C for further chemical analysis. At the end of the incubation, 10 mL of filtered samples were collected for chemical analysis while the rest kept unfiltered for microbiological, molecular and metaproteomic analysis.

**Metaproteomics**

Protein extraction (pellet)

Pellets were resuspended in 2 mL 50 mM Tris HCL pH 6.8. Additionally, 5 g silica beads (0.5 mm zirconia silica beads), 4 mL of a 2 M sucrose solution and 700 mL liquid phenol (10 g phenol in 1 mL water) were added and the cells were lysed in a FastPrep®-24 ball mill (MP Biomedicals GmbH, Eschwege) (5 min, 18000 rpm). To purify proteins, the reaction tube was centrifuged (10 min, 8500 *g*, 4 °C), the upper phenol phase was transferred into a new 50 mL reaction tube and washed with the same volume of 1 M sucrose solution on a thermo mixer (10 min, 60 rpm, room temperature [RT]). After a further centrifugation step (10 min, 10000 *g*, RT) the phenol phase was transferred again into a new 50 mL reaction tube. Then proteins were precipitated twice, once with the four-fold and once with the three-fold volume of ice-cold 0.1 M ammonium acetate in methanol. After each precipitation step the reaction tube was incubated at -20 °C for 1 h and the supernatant was discarded after centrifugation (10 min, 8500 *g*, 4 °C). For removal of remaining impurities, the pellet was washed four times. Therefore, two times the threefold volume of ice-cold 80% acetone (v/v) respectively ice-cold 70% ethanol (v/v) were used alternating. Between each precipitation step the reaction tube was incubated for 15 min at -20 °C and the supernatant was removed after centrifugation (10 min, 8500 *g*, 4 °C). Finally, the pellet was dissolved in 500 to 700 µL urea buffer (7 M urea, 2 M thiourea, 0.01 g mL^-1^ 1,4-dithiothreitol).

Protein quantification

For protein quantification 300 µL amido black dye solution (0.26 mg mL^-1^) was mixed with 50 µL protein extract. Afterwards, the excess dye was removed by centrifugation (16400 *g*, 5 min, RT) and the supernatant discarded, followed by two washing steps with 500 µL 10% methanol (v/v). Between each washing step the samples were centrifuged (16400 *g*, 5 min, RT). Finally, the pellet was dissolved in 1 mL 0.1 M sodium hydroxide solution, the absorption was measure at 615 nm and compared against a standard curve with bovine serum albumin.

SDS-PAGE

To pre-purify samples for MS/MS, proteins were loaded additionally onto a SDS-PAGE (Laemmli, 1970). Therefore 25 µg protein extract were diluted in the same volume of distilled water and precipitated with the five-fold volume of ice-cold 100% acetone. After 1 h incubation at -20 °C and centrifugation (for 30 min at 16400 *g* and 4 °C) the supernatant was discarded and the pellet was dried under a hood. Subsequently, the pellet was dissolved in 21 µL SDS sample buffer during shaking on a thermo mixer (5 min, 1400 rpm, 60 °C). Finally, insoluble material was removed by centrifugation (10 min, 16400 *g*, RT) and the supernatant was loaded on a 1 mm 12% SDS-PAGE (Mini-Protean® Tetra System, BIO-RAD, Hercules, USA). However, SDS-PAGE was stopped after the proteins entered approximately 5 mm into the separation gel. For visualization of the proteins the gel was stained with colloidal coomassie (Neuhoff et al., 1985) and scanned (ViewPix 900 Scanner, Biostep, Burkhardtsdorf, Germany) with 300 dpi.

Tryptic digestion

Tryptic digestion of the complete protein fraction from the SDS-PAGE was carried out as previously described by (Shevchenko et al., 1996). Therefore, the stained protein fraction was cut-off from the SDS-PAGE, chopped into pieces of 1 mm size and transferred into a 2 mL reaction tube. Coomassie dye and other impurities were removed by two washing steps with 900 µL washing solution (50% methanol [v/v], 5% acetic acid [v/v]) (1 h, 150 rpm, RT) and one with acetonitrile (5 min, 150 rpm, RT) on a thermo mixer. After the gel pieces were entirely dried, proteins were denaturated with 900 µL 45 mM 1,4-dithiotreitol (30 min, 150 rpm, RT) and alkylated with 900 µL of a 100 mM iodacetamide (30 min, 150 rpm, RT). After each step the gel pieces were washed with 900 µL acetonitrile (5 min, 150 rpm, RT). Subsequently, two further washing steps one with 25 mM ammonium bicarbonate (10 min, 150 rpm, RT) and one with acetonitrile (5 min, 150 rpm, RT) were carried out. Proteins were digested then by the addition of 200 µL trypsin buffer (overnight, 150 rpm, 37 °C), containing 0.07 µg trypsin dissolved in 25 mM ammonium bicarbonate. The next day the peptides in the supernatant were collected into a new 2 mL reaction tube. Remaining peptides from the gel pieces were extracted by the incubation with 10% (v/v) formic acid as well as with a mixture of 49% acetonitrile and 1% triflouric acid and were also collected into the new 2-mL reaction tube. Finally, the peptides were dried in a speedvac (Digital Series SpeedVac SPD121P, Thermo Scientific, Waltham, USA)

Mass spectrometry

Peptides were analyzed by LC-MS/MS using an UltiMate 3000 RSLCnano splitless liquid chromatography system, coupled online to an Orbitrap Elite™ Hybrid Ion Trap-Orbitrap Mass Spectrometer (both from Thermo Fisher Scientific, Bremen, Germany). After injection, peptides were loaded isocratically on a trap column (Dionex Acclaim, nano trap column, 100 μm i.d. x 2 cm, PepMap100 C18, 5 μm, 100 Å, nanoViper) with a flow rate of 7 μL min^-1^ chromatographic liquid phase A (98% LC-MS Water, 2% ACN, 0.05% TFA) for desalting and concentration.

Chromatographic separation was performed on a Dionex Acclaim PepMap C18 RSLC nano reversed phase column (2 μm particle size, 100 Å pore size, 75 μm inner diameter and 250 mm length) at 40 °C column temperature. A flow rate of 300 nL min^-1^ was applied using a binary A/B-solvent gradient (solvent A: 98% LC-MS Water, 2% acetonitrile, 0.1% formic acid; solvent B: 80% acetonitrile, 10% LC-MS Water, 10% trifluorethanol, 0.1% formic acid) starting with 4% B for 4 min, continuing with a linear increase to 55% B within 120 min, followed by a column wash with 90% B for 5 min and a re-adjusted equilibration with 4% B for 25 min. For MS acquisition a data-dependent MS/MS method was chosen. For the conducted measurements MS was operated in positive ion mode, and precursor ions were acquired in the orbital trap of the hybrid MS at a resolution of 30000 and a m/z range of 350 to 2000. Subsequently, the fragment ion scan was proceeded in the linear ion trap of the hybrid MS with a mass range and a scan rate with “normal” parameter settings for the top 20 most intense precursors selected for collision-induced dissociation.

Bioinformatic data evaluation

MS results were processed by the Proteome Discoverer Software 1.4 (Thermo Fisher Scientific, Bremen, Germany) and exported as mascot generic format (.mgf). For protein identification multiple database searches were conducted using Mascot (version 2.5), X!tandem (version15.12.2015) and OMSSA (versionomssa-2.1.9), whereas for X!Tandem and OMSSA search the .mgf-files were directly imported into and extended version of the MPA Software (https://code.google.com/p/meta-proteome-analyzer/, version 1.3.4) for MASCOT 2.5 the mgf.-files were imported first into the ProteinScape software (Bruker Daltonics, Bremen, Deutschland, version 3.1.3.461) and afterwards the results were uploaded as Mascot result files (.dat-files) into the MPA software.

For all database search algorithms, following search parameters were applied: trypsin, one missed cleavage, monoisotopic mass, carbamidomethylation (cysteine) and oxidation (methionine) as variable modifications, ±10 ppm precursor and ±0.5 Da MS/MS fragment tolerance, 113C and +2/+3 charged peptide ions, 1% false discovery rate. The protein database contained all UniProt/Swiss-Prot entries (version: 14.06.2016), the UniProt Tremble entries for the giant panda (version: 14.06.2016), as well as entries of three metagenomes of panda microbiomes. Entries from metagenome were annotated by a BLAST search (version: ncbi-blast-2.3.0+) against UniProt/Swiss-Prot entries (version: 14.06.2016) with an e-value threshold of 10-4. For the final taxonomic and functional data evaluation redundant protein identifications were grouped to so called metaproteins based at least one shared peptide.

**Molecular analyses**

DNA extraction

DNA extraction for 16S microbial community analysis was conducted on 1 g bamboo material or on the pellet resulting from 2 mL liquid samples, which were centrifuged in a FastPrep tube (5 min, 13000 rpm). Samples were supplied with 200 mg glass beads (0.11 mm, Sartorius) and 1 mL lysis buffer (100 mM Tris, 100 mM EDTA, 100 mM NaCl, 1% polyvinylpyrrolidone [PVP40], 2% sodium dodecyl sulphate [SDS]; pH 8). Tubes were placed in a FastPrep device (MP Biomedicals, USA) (16000 rpm, 40 s, 2 runs), centrifuged (10 min, max speed, 4 °C), the DNA extracted with phenol-chloroform and precipitated with ice-cold isopropyl alcohol and 3 M sodium acetate (1 h, -20 °C). Isopropyl alcohol was removed by centrifugation (30 min, max speed), DNA pellets dried and resuspended in TE buffer (10 mM Tris, 1 mM EDTA) and stored at -20 °C. DNA sample quality was assessed using 1% (w:v) agarose (Life technologiesTM, Spain) gel-electrophoresis, and quantified by a fluorescence assay (QuantiFluor® dsDNA kit; Promega, USA) using a Glomax®-Multi+ system (Promega). Samples were normalized to 1 ng µL^-1^ DNA and sent to LGC Genomics (Germany) for library preparation and sequencing using the Illumina Miseq platform.

Illumina gene sequencing

Briefly, in a first step the 16S rRNA gene V3-V4 hypervariable regions were amplified by PCR using primers derived from (Klindworth et al., 2013), with a modification to the reverse primer by introducing another wobble position (K) to make it more universal. The PCR mix included 1 ng DNA extract, 15 pmol of both the forward primer 341F 5'- NNNNNNNNNTCCTACGGGNGGCWGCAG and reverse primer 785R 5'- NNNNNNNNNNTGACTACHVGGGTATCTAAKCC in 20 µL volume of MyTaq buffer containing 1.5 units MyTaq DNA polymerase (Bioline) and 2 µL of BioStabII PCR Enhancer (Sigma). For each sample, the forward and reverse primers had the same unique 10-nt barcode sequence (represented by (N)10 in the primer sequences). PCRs were carried out for 20 cycles as follows: 2 min 96 °C pre-denaturation; 96 °C for 15 s, 50 °C for 30 s, 70 °C for 90 s. If needed PCRs showing low yields were further amplified for 5 cycles. DNA concentration of amplicons of interest was determined by gel-electrophoresis. About 20 ng amplicon DNA of each sample were pooled for up to 48 samples carrying different barcodes. The amplicon pools were purified with one volume AMPure XP beads (Agencourt) to remove primer dimer and other small mispriming products, followed by additional purification on MinElute columns (Qiagen). Finally, about 100 ng of each purified amplicon pool DNA was used to construct Illumina libraries by means of adaptor ligation using the Ovation Rapid DR Multiplex System 1-96 (NuGEN). Illumina libraries were pooled and size-selected by preparative gel-electrophoresis. Sequencing was performed on an Illumina MiSeq using version 3 chemistry (Illumina).

Illumina analysis

High-throughput amplicon sequencing of the V3-V4 hypervariable region (Klindworth et al., 2013) was performed with the Illumina MiSeq platform according to the manufacturer’s guidelines at LGC Genomics GmbH (Berlin, Germany). Contigs were created by merging paired-end reads based on the Phred quality score (of both reads) heuristic as described by (Kozich et al., 2013) in Mothur (Schloss et al., 2009) (v.1.33.3). Contigs were aligned to the mothur-recreated SILVA SEED alignment, release 123 and filtered from those with (i) ambiguous bases, (ii) more than 8 homopolymers, and (iii) those not corresponding to the V3 – V4 region, retaining 63% of the data. The initial sequences were prescreened removing all sequences with ambiguous base calls, as well as any sequences longer than 450 nucleotides or shorter than 402 nucleotides. The remaining sequences with a length between 402 and 449 bases, were aligned to mothur formatted silva.seed release 123 alignment database trimmed between positions 6388 and 22096 to be compatible with the 341F-785Rmod primers. Any sequences not aligning within this region or containing homopolymer stretches of length more than 8 were removed. In a next step, data was pre-clustered allowing up to 4 differences between sequences to be merged. A chimera check was performed using UCHIME (de novo). Sequences were consequently classified by means of a naïve Bayesian classifier, against the RDP 16S rRNA gene training set, version 10 with an 80% cut-off for the pseudobootstrap confidence score. Taxa with annotation *Chloroplast*, *Mitochondria*, unknown or *Eukaryota* at the kingdom level were excluded. Sequences were clustered into OTUs within each order identified by the preceding classification step. The cut-off used in the cluster.split command was set at 0.15. Eventually we choose to bin sequences in OTUs at a 3% dissimilarity level to generate a contingency table. Finally taxonomy assignment is obtained using the classify.otu command or the get.oturep and classify.seq command according to the RDP version 10 and silva.nr_v123 database. The contingency table, containing the number of reads observed for each OTU in each sample, was loaded into R version 3.2.2 (2015-08-14), running on a GNU/Linux 3.13.0-57-generic x86_64 system. After examining read counts, if any OTU was not classified up to genus level, the consensus sequence was blasted using the NCBI database to obtain the taxonomic classification. Singletons that remained unclassified were culled. Samples were rescaled to 8481 reads for further analysis.

**Microbiological analysis**

Cell count and intact/damaged cell count was performed by flow cytometry at the beginning and at the end of incubations. SYBR® Green I and Propidium Iodide (PI) were used alone and in combination to discriminate cells with intact and damaged cytoplasmic membranes as follows: PI (20 mM in dimethyl sulfoxide [DMSO], LIVE/DEAD BacLight Kit, Invitrogen, Belgium) was diluted 50 times and SYBR® Green I (10000 times concentrate in DMSO, Invitrogen) was diluted 100 times in 0.22 μm-filtered-DMSO. Water samples were stained with 10 μL mL^-1^ staining solution and 10 μL mL^-1^ EDTA (pH 8, 500 mM) for outer membrane permeabilization. Before staining, samples of 1 mL were maintained at room temperature for 30 min to minimize staining temperature effects. Prior to flow cytometric analysis, stained samples were incubated for 13 min in the dark at 37 °C. Flow cytometry was performed using a CyAn™ ADP LX flow cytometer (Dakocytomation, Heverlee, Belgium) equipped with a 50 mW Sapphire solid-state diode laser (488 nm). Stability and performance were performed using the Cyto-Cal Alignment Beads and Cyto-Cal multifluor Fluorescent Intensity Calibrator (Distrilab, Leusden, The Netherlands). Green and red fluorescence were collected with photomultiplier tubes using 530/40 and 613/20 bandpass filters respectively. MilliQ water was used as the sheath fluid. All samples were collected as logarithmic signals triggered on the green fluorescence channel. Data for 20000 events for each sample run was collected.

**Chemical analyses**

Lignocellulose content in bamboo was measured following the Van Soest method (Van Soest, 1963). Between 0.5 and 1 g of dry sample material was treated with cetyl-trimethylammonium-bromide to dissolve and remove proteins. The remaining material was first dried (105 °C) and weighed again, and then treated with a 72% sulphuric acid solution. After again drying (105 °C) and weighing, the cellulose content is calculated by subtracting the mass before and after the sulphuric acid treatment and dividing this value by the initial material mass. In a third step the remaining material was reduced to ash at 550 °C: the lignin content was then calculated by subtracting the mass before and after reducing to ash and dividing it by the initial material mass.

Gas quality composition was analyzed with a Compact GC (Global Analyser Solutions, Breda, The Netherlands), equipped with a Molsieve 5Å pre-column and two channels. In channel 1, a Porabond column detected CH_4_, O_2_, H_2_ and N_2_. In channel 2, an Rt-Q-bond pre-column and column detected CO_2_, N_2_O and H_2_S. Gas concentrations were determined with a thermal conductivity detector. Volatile fatty acids between C_2_-C_8_ (including isoforms C_4_-C_6_) and alcohols were measured by gas chromatography (GC-2014, Shimadzu®, The Netherlands) with DB-FFAP 123-3232 column (30 m x 0.32 mm x 0.25 µm; Agilent, Belgium) and a flame ionization detector (FID). Liquid samples were conditioned with sulfuric acid and sodium chloride and 2-methyl hexanoic acid as internal standard for quantification of further extraction with diethyl ether. Prepared sample (1 µL) was injected at 200 ºC with a split ratio of 60 and a purge flow of 3 mL min^-1^. Oven temperature increased by 6 ºC min^-1^ from 110 to 165 ºC where it was kept for 2 min. FID had a temperature of 220 ºC. The carrier gas was N_2_ at a flow rate of 2.49 mL min^-1^.

Lactic and formic acid concentrations were determined with a 930 Compact IC Flex (Metrohm, Switzerland) ion chromatography (IC) system with inline bicarbonate removal (MCS), equipped with a guard column cartridge (Metrosep Dual 4/4.6, Metrhom) and an organic acids column (Metrosep 250/7.8, Metrohm) with a 850 IC conductivity detector. Oven temperature was set at 35 ºC. A 1 mM H_2_SO_4_ solution was used as eluent at a flow rate of 0.5 mL min^-1^. Alcohols including glycerol and ethanol were determined with the same IC equipped with a guard column cartridge (Metrosep Trap 1 100/4.0, Metrohm) and an alcohols column (Metrosep Carb 2 250/4.0, Metrohm) with an IC amperometric detector. Oven temperature was set at 35 ºC. A 20 mM NaOH solution was used as eluent at a flow rate of 0.8 mL min^-1^.

**Statistical analysis**

All statistical analyses were performed in the R statistical environment (v3.5.1) (Team, 2015), using functions from the phyloseq (v1.16.2), DESeq2 (v1.22.1) and Phenoflow (v1.1) packages (McMurdie and Holmes, 2013; Love et al., 2014). Errors on all summary statistics represent standard deviations on the mean and were calculated by propagating individual standard deviations as randomly distributed, independent errors. Alpha diversity was assessed by the Hill diversity numbers, which incorporate both richness and evenness components (Hill, 1973). For calculating the alpha diversity, we used the Diversity_16S function from the Phenoflow package. For each sample we generated 100 bootstrap samples, took the average diversity (Hill order 2 which equals the Inverse Simpson index) as the sample representative diversity. For beta diversity analysis the taxon abundances were rescaled by calculating their proportions and multiplying them by the minimum sample size present in the data set (McMurdie and Holmes, 2014). The beta diversity was then assessed by Principal Coordinate analysis (PCoA) of the Bray-Curtis dissimilarity matrix.

**References**

Hill, M.O. (1973). Diversity and evenness: a unifying notation and its consequences. *Ecology* 54(2)**,** 427-432.

Klindworth, A., Pruesse, E., Schweer, T., Peplies, J., Quast, C., Horn, M., et al. (2013). Evaluation of general 16S ribosomal RNA gene PCR primers for classical and next-generation sequencing-based diversity studies. *Nucleic Acids Res* 41(1)**,** e1. doi: 10.1093/nar/gks808.

Kozich, J.J., Westcott, S.L., Baxter, N.T., Highlander, S.K., and Schloss, P.D. (2013). Development of a dual-index sequencing strategy and curation pipeline for analyzing amplicon sequence data on the MiSeq Illumina sequencing platform. *Appl Environ Microbiol* 79(17)**,** 5112-5120. doi: 10.1128/AEM.01043-13.

Laemmli, U.K. (1970). Cleavage of structural proteins during the assembly of the head of bacteriophage T4. *Nature* 227(5259)**,** 680-685.

Li, Y., Guo, W., Han, S., Kong, F., Wang, C., Li, D., et al. (2015). The evolution of the gut microbiota in the giant and the red pandas. *Sci Rep* 5**,** 10185. doi: 10.1038/srep10185.

Love, M.I., Huber, W., and Anders, S. (2014). Moderated estimation of fold change and dispersion for RNA-seq data with DESeq2. *Genome Biol* 15(12)**,** 550. doi: 10.1186/s13059-014-0550-8.

McMurdie, P.J., and Holmes, S. (2013). phyloseq: an R package for reproducible interactive analysis and graphics of microbiome census data. *PLoS One* 8(4)**,** e61217. doi: 10.1371/journal.pone.0061217.

McMurdie, P.J., and Holmes, S. (2014). Waste Not, Want Not: Why Rarefying Microbiome Data Is Inadmissible. *Plos Computational Biology* 10(4).

Neuhoff, V., Stamm, R., and Eibl, H. (1985). Clear Background and Highly Sensitive Protein Staining with Coomassie Blue Dyes in Polyacrylamide Gels - a Systematic Analysis. *Electrophoresis* 6(9)**,** 427-448. doi: DOI 10.1002/elps.1150060905.

Schloss, P.D., Westcott, S.L., Ryabin, T., Hall, J.R., Hartmann, M., Hollister, E.B., et al. (2009). Introducing mothur: Open-Source, Platform-Independent, Community-Supported Software for Describing and Comparing Microbial Communities. *Applied and Environmental Microbiology* 75(23)**,** 7537-7541. doi: 10.1128/Aem.01541-09.

Shevchenko, A., Wilm, M., Vorm, O., and Mann, M. (1996). Mass spectrometric sequencing of proteins silver-stained polyacrylamide gels. *Anal Chem* 68(5)**,** 850-858.

Tabet, R.B., Oftedal, O.T., and Allen, M.E. (Year). "Seasonal differences in composition of bamboo fed to giant pandas (Ailuropoda melanoleuca) at the National Zoo", in: *Proceedings of the Fifth Comparative Nutrition Society Symposium*: Hickory Corners (MI, USA)), 176-183.

Team, R.C. (2015). *A language and environment for statistical computing.* R Foundation for Statistical Computing.

Van Soest, P.J. (1963). Use of detergents in the analysis of fibrous feeds. II. A rapid method for the determination of fiber and lignin. *Journal of the A.O.A.C* 45(5)**,** 829-835.

Williams, C.L., Dill-McFarland, K.A., Vandewege, M.W., Sparks, D.L., Willard, S.T., Kouba, A.J., et al. (2016). Dietary Shifts May Trigger Dysbiosis and Mucous Stools in Giant Pandas (Ailuropoda melanoleuca). *Front Microbiol* 7**,** 661. doi: 10.3389/fmicb.2016.00661.

Xue, Z., Zhang, W., Wang, L., Hou, R., Zhang, M., Fei, L., et al. (2015). The bamboo-eating giant panda harbors a carnivore-like gut microbiota, with excessive seasonal variations. *MBio* 6(3)**,** e00022-00015. doi: 10.1128/mBio.00022-15.

Zhang, W., Liu, W., Hou, R., Zhang, L., Schmitz-Esser, S., Sun, H., et al. (2018). Age-associated microbiome shows the giant panda lives on hemicelluloses, not on cellulose. *ISME J* 12(5)**,** 1319-1328. doi: 10.1038/s41396-018-0051-y.
